# Digital biomarkers for brain health: passive and continuous assessment from wearable sensors

**DOI:** 10.1038/s41746-026-02340-y

**Published:** 2026-01-14

**Authors:** Igor Matias, Maximilian Haas, Eric J. Daza, Matthias Kliegel, Katarzyna Wac

**Affiliations:** 1 Quality of Life Technologies Lab, University of Geneva, Geneva, Switzerland; 2 Cognitive Aging Lab, University of Geneva, Geneva, Switzerland; 3 Faculty of Psychology, UniDistance Suisse, Geneva, Switzerland; 4 Stats-of-1, Menlo Park, California, United States of America; 5 Boehringer Ingelheim Pharmaceuticals Inc., Ridgefield, California, United States of America

**Keywords:** Biomarkers, Health care, Neuroscience

## Abstract

Continuous and scalable monitoring of cognition and affective states is critical for the early detection of brain health, which is currently limited by the burden of active assessments. This study investigated the potential of consumer-grade wearable and mobile technologies to passively predict 21 cognitive and mental health outcomes in real-world conditions. We collected data from 82 cognitively healthy adults, including passively measured behaviour, physiology, and environmental exposures longitudinally, for 10 months. Active data were gathered in four waves using validated patient- and performance-reported outcomes. Data quality assurance involved a data filtering resulting in average wearable data coverage of 96% per day. Artificial Intelligence-powered prediction was applied, and performance was assessed using subject- and wave-dependent cross-validation. Cognitive and affective outcomes were predicted with low scaled errors. Patient-reported outcomes were more predictable than performance-based ones. Environmental and physiological metrics emerged as the most informative predictors. Passive multimodal data captured meaningful variability in cognition and affect, demonstrating the feasibility of low-burden, scalable approaches to continuous brain-health monitoring. Feature-importance analyses suggested that environmental exposures better explained inter-individual differences, whereas physiological and behavioural rhythms captured within-person changes. These findings highlight the potential of everyday technologies for population-level tracking of brain-health and deviations from expected trajectories.

## Introduction

Understanding how cognition and affect fluctuate in daily life is central to promoting lifelong brain health—here defined as the combination of cognitive performance and affective states^1–3^. Even among healthy adults, brain health varies across days and weeks, reflecting interactions between several factors^4^. Yet, most assessments remain episodic and burdensome, limiting their ability to capture high-frequency and natural dynamics, provide timely feedback, or scale to population-level monitoring^1,5,6^.

Mobile and wearable technologies now enable passive, continuous, and ecologically valid data collection in real-world settings^7^. These tools allow frequent observation of everyday behaviour, physiology, and environmental context—marking a shift from laboratory or questionnaire-based methods toward scalable, low-burden approaches that can reveal meaningful intra-individual change over time^8^. Continuous monitoring of such cognitive and affective “fluctuations”^1^ may help establish baselines, detect deviations from expected trajectories, and support earlier identification of at-risk states for cognitive and mental disorders.^1^.

Everyday exposures and lifestyle factors—including sleep, physical activity, and environmental conditions—are closely linked to both short- and long-term brain health. Sleep fragmentation and related parameters correlate with cognitive performance, *Alzheimer’s disease* (AD), and affect^9–11^; heart rate and activity patterns relate to cognitive and affective states^9,12^; and exposure to air pollutants and weather variations influences cognition and affect^13–16^. The growing burden of age-related cognitive decline underscores the urgency of developing scalable, preventive monitoring strategies. AD, the leading cause of dementia, already affects tens of millions worldwide and lacks curative treatment^17^. Early detection and preventive monitoring are therefore crucial to preserving independence and quality of life and facilitating access to emerging disease-modifying interventions^18,19^.

In cognitively healthy individuals, capturing natural variability rather than pathology provides a baseline for understanding how everyday physiology and context shape brain-health trajectories before measurable decline^1,4^. Such population-based monitoring complements diagnostic studies in clinical cohorts and advances medicine “intended to screen, track, or treat” changes before disease onset^20^.

While digital biomarkers have gained growing attention^21^, the combination of multiple domains of passively collected data—behavioural, physiological, and environmental—remains limited in studies predicting cognitive and affective functioning in healthy adults. Previous studies have focused on single predictors, limited outcomes, or short observation periods. For instance, Zhou et al.^22^ distinguished cognitive frailty from healthy status using only gait data and a short window of time; Butler et al.^20^ classified mild cognitive impairment using wearable data but did not explore broader markers of brain health; Exler et al.^23^ predicted daily valence from short-term heart-function data; and Cormack et al.^24^ combined smartwatch-based affect and cognition measures without leveraging passive physiological streams. Hickman et al.^25^ further highlighted the need for standardized measures, longer study durations, and consistent analytic frameworks.

Collectively, these efforts underscore an important gap: the integrated, multimodal prediction of everyday cognitive and affective functioning from passively and ubiquitously collected data remains largely unexplored in healthy, community-dwelling adults. Addressing this gap requires longitudinal, multimodal datasets capable of capturing subtle intra-individual changes across behavioural, physiological, and environmental domains. Predictive modelling provides a framework to quantify how strongly such digital signals reflect cognitive and affective fluctuations over time, offering a way to evaluate their utility as digital proxies of brain health and not only certain components of cognition or affective states.

This study addresses that gap using data from the Providemus alz project^26^, a longitudinal, fully remote study combining continuous passive wearable and mobile sensing with quarterly (to balance data frequency with participant burden) active cognitive and affective assessments in healthy adults. We integrated multimodal data over ten months to test how passive behavioural, physiological, and environmental features predict multiple brain-health outcomes. The main goal is to evaluate the feasibility of scalable, low-burden models for continuous brain-health monitoring. This approach lays the groundwork for population-level levels that shift the paradigm from reactive diagnosis to proactive prevention of cognitive and affective decline. We hypothesized that passive multimodal features—particularly those representing sleep, heart rate, and activity—would predict cognitive and affective outcomes with low error rates across individuals.

## Results

### Data quality and outcome models

Due to high missingness, “AQI” and “time zone difference” were excluded, leaving 38 passive predictors across 21 active outcomes. On average, modelling was done with 213.84 ± 3.18 samples for Levels, 130.60 ± 2.90 for ΔABS, and 48.63 ± 7.11 for Δ%. Feature selection ratios and sample sizes are detailed in Tables S3–S4 (Supplementary Materials). The mean daily wear time per day exceeded 96% in all waves. Data quality, preliminary temporal dynamics, and other details are provided in the Supplementary Materials C 1-3. Table 1 and Table 2 report the best results considering the mean SMAE.

**Table 1:** Lowest Mean SMAE of each outcome’s model on the second round, with user-dependent CV

Mean SMAE – User-dependent Cross Validation (maximum 82 folds)					
	**Outcome**	**Lowest SMAE** ± **SD**	**Model**	**Representation**	**Feature Selection**
Cognition	Attention*	15.32% ± 21.67%	SVM	Levels	Spearman
	Cognitive decline	3.22% ± 2.15%	SVM	ΔABS	Pearson
	Cognitive flexibility*	14.98% ± 15.59%	SVM	Levels	Pearson
	Inductive reasoning*	13.45% ± 7.28%	SVM	Levels	Pearson
	Inhibitory control*	7.51% ± 8.36%	SVM	Levels	Kendall
	Long-term memory*	16.39% ± 7.24%	XGBoost	Levels	Pearson
	Memory	6.45% ± 5.11%	RF	ΔABS	Spearman
	Processing speed*	19.06% ± 21.26%	SVM	Levels	Pearson
	Prospective memory*	15.14% ± 15.64%	SVM	ΔABS	Kendall
	Short-term memory*	16.41% ± 7.40%	SVM	Levels	Spearman
	Tapping speed*	10.13% ± 9.39%	SVM	ΔABS	Kendall
	Typing speed*	9.57% ± 7.31%	RF	ΔABS	Kendall
	Verbal fluency 1*	14.03% ± 10.72%	XGBoost	ΔABS	Kendall
	Verbal fluency 2*	25.33% ± 18.23%	LightGBM	Δ%	Pearson
	Working memory*	15.36% ± 13.93%	SVM	ΔABS	Pearson
Affective state	Anxiety	7.30% ± 4.49%	LightGBM	ΔABS	Spearman
	Depression	7.78% ± 4.57%	SVM	ΔABS	Pearson
	Hostility	12.68% ± 11.48%	SVM	ΔABS	Pearson
	Negative affect	9.88% ± 8.36%	SVM	ΔABS	Pearson
	Positive affect	12.14% ± 4.59%	SVM	Δ%	Pearson
	Stress	8.10% ± 5.70%	SVM	ΔABS	Kendall

PerfROs are marked with*

**Table 2:** Lowest Mean SMAE of each outcome’s model on the second round, with wave-dependent CV

Mean SMAE – Wave-dependent Cross Validation (maximum 4 folds)					
	**Outcome**	**Lowest SMAE** ± **SD**	**Model**	**Representation**	**Feature Selection**
Cognition	Attention*	14.21% ± 3.12%	SVM	Levels	Spearman
	Cognitive decline	3.19% ± 0.16%	SVM	ΔABS	Pearson
	Cognitive flexibility*	12.84% ± 3.64%	SVM	Levels	Pearson
	Inductive reasoning*	12.79% ± 1.61%	SVM	Levels	Kendall
	Inhibitory control*	7.38% ± 0.37%	SVM	Levels	Kendall
	Long-term memory*	16.08% ± 1.31%	SVM	Levels	Kendall
	Memory	7.76% ± 0.26%	SVM	ΔABS	Pearson
	Processing speed*	16.47% ± 1.66%	SVM	Levels	Pearson
	Prospective memory*	16.39% ± 2.89%	SVM	Levels	Spearman
	Short-term memory*	14.39% ± 1.68%	SVM	Levels	Spearman
	Tapping speed*	9.75% ± 0.50%	SVM	ΔABS	Spearman
	Typing speed*	9.75% ± 0.17%	SVM	ΔABS	Pearson
	Verbal fluency 1*	13.40% ± 0.83%	SVM	Levels	Spearman
	Verbal fluency 2*	25.06% ± 5.22%	SVM	Δ%	Spearman
	Working memory*	16.67% ± 1.58%	SVM	Levels	Pearson
Affective state	Anxiety	7.68% ± 0.06%	SVM	ΔABS	Spearman
	Depression	8.33% ± 1.05%	SVM	ΔABS	Pearson
	Hostility	9.08% ± 0.48%	SVM	ΔABS	Spearman
	Negative affect	9.38% ± 0.06%	SVM	ΔABS	Kendall
	Positive affect	11.59% ± 1.27%	SVM	ΔABS	Spearman
	Stress	8.70% ± 1.20%	SVM	ΔABS	Pearson

PerfROs are marked with *

**Fig. 1:**
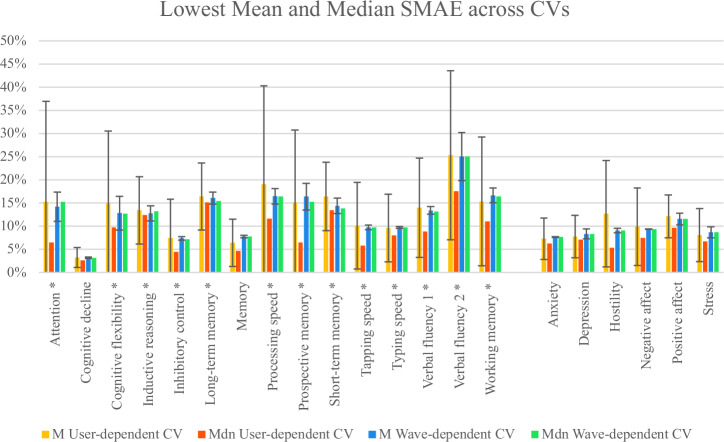
Comparison of the models’ lowest SMAE using both CVs but considering the Mean (M ± SD) or the Median (Mdn) of the results. PerfROs are marked with *. Dispersion measures (IQR and min–max) are reported in Tables 5–6 but not illustrated here to avoid mixing different variability metrics within the same plot.

The following two tables (Table 3 and Table 4) present the same results using median SMAE across users and waves, to help understand the influence of outlier performance (i.e., extreme SMAE values). To complement them and convey variability, we report the IQR for user-dependent CV, which includes up to 82 folds, and the minimum–maximum range for wave-dependent CV, which includes up to four folds. Figure 1 compares the lowest SMAE based on mean and median results for both CVs.

**Table 3:** Lowest Median SMAE of each outcome’s model on the second round, with user-dependent CV

Median SMAE – User-dependent Cross Validation (maximum 82 folds)					
	**Outcome**	**Lowest SMAE (IQR)**	**Model**	**Representation**	**Featue Selection**
Cognition	Attention*	6.46% (12.84%)	SVM	Levels	Kendall
	Cognitive decline	2.63% (3.04%)	LightGBM	ΔABS	Spearman
	Cognitive flexibility*	9.74% (8.64%)	SVM	Levels	Pearson
	Inductive reasoning*	12.44% (10.36%)	SVM	Levels	Pearson
	Inhibitory control*	4.45% (4.57%)	SVM	Levels	Kendall
	Long-term memory*	15.16% (9.98%)	XGBoost	Levels	Pearson
	Memory	4.70% (6.18%)	RF	ΔABS	Spearman
	Processing speed*	11.62% (11.85%)	SVM	Levels	Pearson
	Prospective memory*	6.53% (21.28%)	SVM	ΔABS	Kendall
	Short-term memory*	13.50% (14.89%)	RF	ΔABS	Pearson
	Tapping speed*	5.85% (4.90%)	SVM	Δ%	Kendall
	Typing speed*	8.01% (9.47%)	RF	ΔABS	Kendall
	Verbal fluency 1*	8.81% (14.54%)	SVM	Δ%	Pearson
	Verbal fluency 2*	17.57% (46.69%)	RF	Δ%	Pearson
	Working memory*	11.06% (12.16%)	SVM	ΔABS	Kendall
Affective state	Anxiety	6.27% (6.01%)	LightGBM	ΔABS	Spearman
	Depression	7.07% (5.68%)	SVM	ΔABS	Pearson
	Hostility	5.38% (12.25%)	RF	ΔABS	Kendall
	Negative affect	7.50% (7.49%)	SVM	ΔABS	Pearson
	Positive affect	9.65% (7.25%)	RF	ΔABS	Pearson
	Stress	6.70% (6.00%)	SVM	ΔABS	Kendall

PerfROs are marked with *

**Table 4:** Lowest Median SMAE of each outcome’s model on the second round, with wave-dependent CV

Median SMAE – Wave-dependent Cross Validation (maximum 4 folds)					
	**Outcome**	**Lowest SMAE (min, max)**	**Model**	**Representation**	**Feature Selection**
Cognition	Attention*	15.29% (10.69, 16.66)	SVM	Levels	Spearman
	Cognitive decline	3.19% (3.07, 3.31)	SVM	ΔABS	Pearson
	Cognitive flexibility*	12.70% (9.28, 16.55)	SVM	Levels	Pearson
	Inductive reasoning*	13.21% (11.17, 14.57)	SVM	Levels	Pearson
	Inhibitory control*	7.20% (7.15, 7.81)	SVM	Levels	Kendall
	Long-term memory*	15.46% (15.19, 17.58)	SVM	Levels	Kendall
	Memory	7.76% (7.70, 8.06)	SVM	ΔABS	Pearson
	Processing speed*	16.44% (14.83, 18.15)	SVM	Levels	Pearson
	Prospective memory*	15.25% (14.25, 19.67)	SVM	Levels	Spearman
	Short-term memory*	13.83% (13.46, 16.63)	SVM	Levels	Kendall
	Tapping speed*	9.75% (9.40, 10.11)	SVM	ΔABS	Spearman
	Typing speed*	9.75% (9.63, 9.87)	SVM	ΔABS	Pearson
	Verbal fluency 1*	13.16% (12.77, 15.36)	SVM	Levels	Pearson
	Verbal fluency 2*	25.06% (21.37, 28.76)	SVM	Δ%	Spearman
	Working memory*	16.45% (15.22, 18.35)	SVM	Levels	Pearson
Affective state	Anxiety	7.68% (7.65, 7.72)	SVM	ΔABS	Spearman
	Depression	8.33% (7.59, 9.07)	SVM	ΔABS	Pearson
	Hostility	9.08% (8.74, 9.42)	SVM	ΔABS	Spearman
	Negative affect	9.38% (9.34, 9.43)	SVM	ΔABS	Kendall
	Positive affect	11.59% (10.69, 12.48)	SVM	ΔABS	Spearman
	Stress	8.70% (7.80, 9.50)	SVM	ΔABS	Pearson

PerfROs are marked with *

To further evaluate the meaningfulness of the obtained prediction errors, we compared the models’ performance (mean SMAE using user-dependent CV) against a naïve population mean predictor, which used the grand mean of each outcome across all participants and all the measurement waves as the predicted value. This constant predictor ignores temporal or inter-individual variability and therefore serves as a reference for assessing the added value of the machine-learning models. For scientific rigor, the naïve model’s performance was computed using the same data representations as the corresponding machine-learning models, ensuring comparable variability and value ranges.

In addition to inspecting SMAE differences, we conducted formal statistical tests to determine whether the model improvements were larger than would be expected by random variation. For each outcome, paired fold-level errors (model MAE vs. naïve MAE) were compared after assessing normality of the paired differences (Shapiro–Wilk test). When normality was met, we applied a paired t-test; otherwise, we used the Wilcoxon signed-rank test.

As shown in Table S5 of the Supplementary Materials, statistical comparisons revealed that the proposed models achieved significantly lower error than the naïve baseline for three outcomes, all PerfROs—attention, cognitive flexibility, and verbal fluency 1 (all p < 0.05). For one PRO, hostility, the naïve predictor performed significantly better than the model (p < 0.05), a pattern consistent with the low variability and narrow dynamic range observed for this construct (Supplementary Table S14). For the remaining 17 outcomes, no statistically significant differences were detected between model and naïve predictions, indicating that the available sample size and fold-level errors may not have provided sufficient statistical power to detect small performance differences. Importantly, for nearly all outcomes—including those without statistically significant differences—the model still produced equal or lower variability (SD) in prediction error relative to the naïve baseline. These findings suggest that while clear significant improvements were observed for a subset of cognitive outcomes, larger datasets may be required to detect more subtle performance gains across the broader set of constructs.

Additionally, to further ensure the robustness of these findings and to exclude any subtle methodological biases, we examined the stability of the feature selection process across individuals. Since the first modelling round served to identify the most predictive features for the second-round models, we verified whether this population-based feature selection could have introduced subtle data leakage between training and test sets. Applying fold-dependent feature selection within the first modelling phase was not feasible in our case, as it would yield different feature sets across folds and thus prevent consistent comparison and interpretation across participants—it would effectively result in separate, participant-specific models rather than a population-level model per outcome. To ensure that the population-level feature selection used to define the second-round models was not biased by potential data leakage or dominated by individual participants, we conducted a leave-one-out stability analysis. For each outcome, we compared the set of features selected when all participants were included (population-selected features) with the sets obtained when each participant was excluded in turn. The overlap and Jaccard indices (Tables S6–S7 in the Supplementary Materials) quantify how consistently the same predictors were retained across these iterations. Both metrics yielded near-perfect stability (mean Overlap > 0.97; mean Jaccard > 0.95 across outcomes), demonstrating that the features identified as most predictive in the first modelling round were highly robust to the exclusion of individual participants. These findings support the validity of the feature selection procedure and confirm that any subtle data leakage effects are unlikely to have influenced the second-round modelling results.

### Lowest prediction errors

**Fig. 2:**
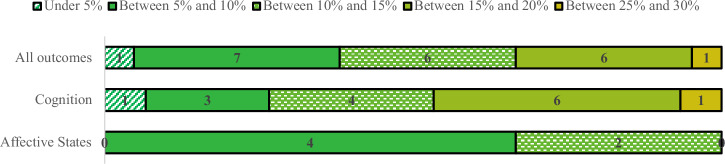
Number of active data outcomes grouped by size of prediction error rate, for all, cognition, and affective states outcomes. Considering only the user-dependent CV M SMAE.

Considering the M SMAE resulting from the user-dependent CV, all the 21 outcomes were predictable with error rates between 3.22% and 25.33%, which show a relevant potential of passive technologies for assessing brain health. As Fig. 2 shows, most cognition models had error rates between 10% and 20%, while that range was between 5% and 10% for the affective states.

**Fig. 3:**
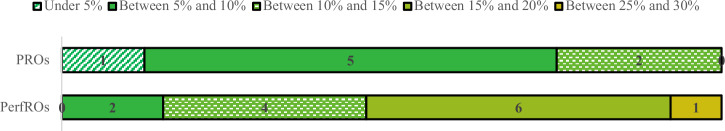
Number of active data outcomes grouped by size of prediction error rate, per type (PRO or PerfRO), for all outcomes combined. Considering only the user-dependent CV M SMAE.

However, as in Fig. 3, only one PRO was predictable under 5% error rate, cognitive decline; PerfROs rates ranged between 10% and 30%, the lowest for inhibitory control. Additionally, all PROs had SMAEs lower than 15%, what did not happen for PerfROs; that may indicate that self-reported outcomes were more predictable than performance-based ones.

### Most predictive passive metrics

**Fig. 4:**
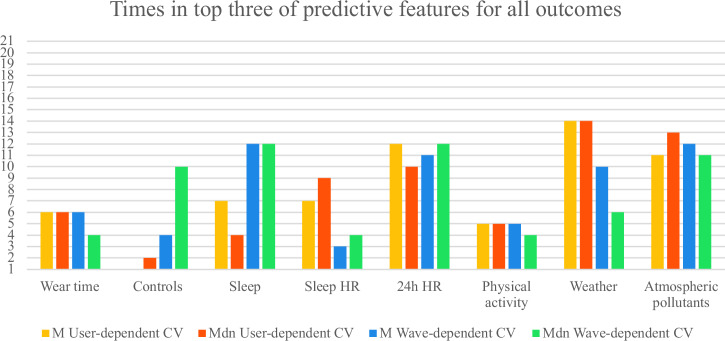
Number of times each group of passive metrics was in the most predictive top three groups across all outcomes. The maximum count possible is 21, the total number of active outcomes.

**Fig. 5:**
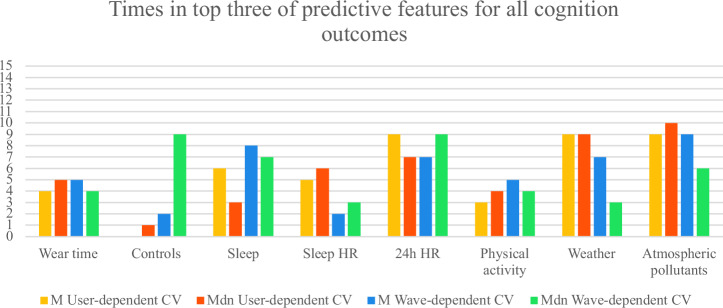
Number of times each group of passive metrics was in the most predictive top three groups across cognition outcomes. The maximum count possible is 15, the number of cognition outcomes.

**Fig. 6:**
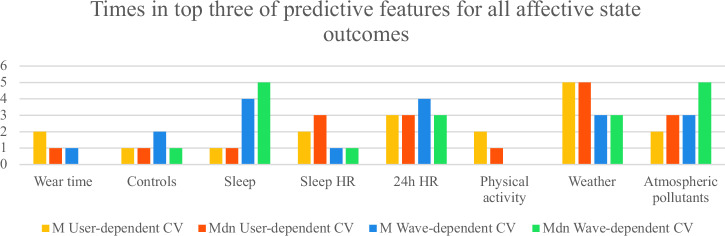
Number of times each group of passive metrics was in the most predictive top three groups across affective state outcomes. The maximum count possible is 6, the number of affective state outcomes.

To identify the most predictive passive metric groups, Fig. 4, Fig. 5, and Fig. 6 show how often each group appeared among the top three predictors for all and each group of outcomes. The scores were adjusted by dividing them by the number of passive metrics in each group. We averaged results across M and Mdn SMAE for both CV types. Overall, excluding control features:

1. For all outcomes, the most predictive groups in user-dependent CV were weather, atmospheric pollutants, and 24 h HR; in wave-dependent CV, sleep, 24 h HR, and atmospheric pollutants.
2. For cognition, top predictors were atmospheric pollutants, weather, and 24 h HR (user-dependent); 24 h HR, sleep, and atmospheric pollutants (wave).
3. For affective states, user-dependent CV highlighted weather, 24 h HR, sleep HR, and atmospheric pollutants; wave-dependent pointed to sleep, atmospheric pollutants, and 24 h HR.

These patterns are summarized in Table 5, which highlights the top passive data groups across cognitive and affective domains for both user- and wave-dependent CV.

**Table 5:** Overview of the most predictive passive data groups across outcomes and CV schemes

	All outcomes		Cognition		Affective states	
**Passive data group**	User-dependent CV	Wave-dependent CV	User-dependent CV	Wave-dependent CV	User-dependent CV	Wave-dependent CV
Weather	**X**		**X**		**X**	
Atm. pollutants	**X**	**X**	**X**	**X**		**X**
24 h HR	**X**	**X**	**X**	**X**	**X**	**X**
Sleep		**X**		**X**		**X**
Sleep HR					**X**	

Each “X” indicates that the corresponding passive data group ranked among the top three most predictive groups for the given outcome type and CV approach (user-dependent or wave-dependent). The results are based on averaged M and Mdn SMAE scores normalized by the number of metrics per group

Figures S5–S8 (Supplementary Materials) display the distribution of feature importance across passive data metric groups. To further enhance interpretability, we summarized the three most informative predictors for each of the 21 outcomes based on the mean feature importance values obtained from the model with the lowest SMAE under user-dependent CV. As presented in Table S8 of the Supplementary Materials, HR- and environmental–related predictors (e.g., 24-h HR SD, carbon monoxide, and nitrogen dioxide) were the most recurrently important for cognitive outcomes, whereas physical activity and environmental variables (e.g., active calories burned, air temperature minimums, and sulfur dioxide) appeared more frequently among affective outcomes. A detailed analysis of these feature-importance patterns, including their temporal dynamics and inter-feature interactions, is beyond the scope of this manuscript and will be the focus of a subsequent publication.

## Discussion

The study analysed 82 cognitively healthy adults from Switzerland and France, providing 10 months of passive data and four waves (repeated assessments) of 21 metrics of cognition and affective states. Because of the inclusion of only healthy subjects, the models developed quantify population-level variability in everyday cognition and affect and are not intended or evaluated as diagnostic tools. Despite its results, this study has limitations. The predominantly Western, Educated, Industrialized, Rich, and Democratic ^27^ participants limit generalizability. The recruitment methods may have favoured digitally literate and cognitively higher-functioning individuals. About 25% of participants completed active assessments in a non-native language, potentially affecting accuracy. Some everyday cognition or affect may have been unmeasured, and PROs could be influenced by social desirability bias. Lastly, we relied on daily summaries. As we prioritized model understandability over predictive power, we didn’t use finer-grained hourly and minute-level data points in our analyses. Such an approach might have revealed higher-resolution patterns, some of which may encounter broader patterns, as in Simpson’s paradox. Future research should replicate these findings in larger, more demographically and clinically diverse samples, including populations at risk for cognitive decline, mild cognitive impairment, dementia, or depression, to evaluate the generalizability and clinical relevance of the proposed models.

In summary, we examined the predictive power of consumer-grade mobile and wearable technologies for predicting fluctuations in everyday cognition and affect, essential for an early detection of brain health issues. We combined 10 months of Intensive Longitudinal Data^28^ passively collected next to 82 cognitively healthy adults with active data from four waves of assessments of their cognitive performance and affective states.

To ensure methodological robustness, we systematically evaluated eight data summaries features, three wave-based aggregation methods, three correlation coefficients to select features, and four machine learning models for outcome prediction. Our passive dataset covered more than 96% of the day per participant.

The low error rates (as low as 3.22% of SMAE) when predicting cognition and affective states outcomes suggest a strong potential for unobtrusively monitoring brain health in daily life. These further underscore the predictive power of passively recorded real-world behavioural and environmental data for measuring underlying cognitive and affective dynamics. PROs were consistently more predictable than PerfROs, reflecting their greater temporal stability, and potentially a stronger sensitivity to environmental and internal states. A possible explanation for this pattern was that PerfROs showed higher within-person variability across waves, which likely contributed to their lower predictability relative to PROs. User-dependent CV yielded lower median error rates than wave-dependent CV, although this advantage was not found when considering mean errors—likely due to between-participant variability and outliers. This highlights the challenges of model generalization across individuals versus time points.

Feature-importance analyses suggested that, for all outcomes of cognition and affect combined, environmental features contributed more to between-person generalization, whereas behavioural and rhythm-related (circadian) measures were more predictive of within-person changes over time. This pattern aligns with existing literature: exposure to weather conditions and atmospheric pollutants is known to influence long-term brain-health trajectories^13^, shaping inter-individual baselines of cognitive and affective functioning (user-dependent CV). Once such baselines are established, relatively stable or cyclic environmental exposure leaves within-individual variability to be driven primarily by physiological and behavioural factors—such as HR dynamics and sleep patterns—which modulate short-term cognitive and affective fluctuations (wave-dependent CV).

Separating the outcomes into cognition and affect, in wave-dependent CV, the top predictive feature groups were consistent for both cognition and affect—namely, atmospheric pollutants, 24-hour HR, and sleep. However, user-dependent CV revealed partially distinct profiles: cognition models were more informed by weather, atmospheric pollutants, and 24-hour HR, whereas affective states were more strongly associated with weather, 24-hour HR, and HR features specifically derived during sleep episodes. Taken together, these patterns are biologically plausible. First, the prominence of atmospheric pollutants for cognition is consistent with evidence that chronic exposure can impair cognitive performance via neuroinflammatory and vascular pathways^14^. While pollutants also relate to affective states, those associations are often nonlinear and heterogeneous across pollutants and time scales, which can make them less uniformly predictive in population models compared with their more consistent cognitive links^15,16^. In other words, the cognitive impact of pollution may present as a steadier, exposure–dose signal, whereas affective responses may depend more on thresholds, susceptibilities, and context—patterns that our user-dependent results likely captured. Second, the specificity of sleep-episode HR features for affect coheres with work showing that disturbed nocturnal autonomic regulation amplifies emotional reactivity and blunt emotion recognition and regulation the following day^10,11^. By contrast, autonomic indices relate to cognition most robustly for select executive components rather than broad cognitive performance, suggesting a more targeted link between cardiac vagal control and cognition^12^. This helps explain why 24-hour HR features (a diurnal autonomic “load” signal) contributed across both domains, while sleep HR emerged as a stronger between-person discriminator for affective profiles but added little to within-person affect dynamics over quarterly waves.

Conceptually, the wave-dependent overlap of sleep, 24-hour HR, and pollutants in both cognition and affect implies a shared within-person substrate: fluctuations in recovery (sleep/autonomic balance) and ambient load (pollution) that modulate day-to-day functioning across domains. Meanwhile, the user-dependent divergence—with sleep HR distinguishing affective profiles and pollutants marking cognitive variability—suggests that trait-like autonomic reactivity during sleep separates individuals in their emotional regulation capacity, whereas ambient exposures better map stable between-person differences in cognitive efficiency.

These interpretations remain associational rather than causal and likely depend on nonlinearities and thresholds (especially for affect and pollution^15^). Still, the converging pattern across CV schemes offers a coherent framework: ambient load helps define who differs from whom (between-person), while endogenous rhythm and recovery help explain how the same person changes (within-person)—with sleep-phase physiology preferentially indexing affective, rather than cognitive, profiles.

Unlike traditional assessments, this framework supports continuous, individualized monitoring in real-world contexts, enabling earlier anomaly detection and more nuanced tracking of brain health trajectories. Clinically, these models can serve as low-burden and constant triage tools for early cognitive or affective disorders signalling, enabling timely medical/expert-based interventions. They’re particularly valuable in primary care and telemedicine, enhancing routine follow-up and relapse prevention without adding clinical workload. These methods also aid in identifying early-stage cases for research or trials and provide a starting point for digital endpoints that reflect everyday function.

This approach democratizes access to brain health tools by relying on widely available, low-cost devices. It paves the way for scalable population-level screening and shifts care from reactive to preventive. Although lacking long-term validation, the results show promise for passively collected digital biomarkers in shaping future brain health care.

Beyond feasibility, practical implementation of continuous digital monitoring requires addressing some translational challenges. First, privacy and data security are essential. By emphasizing model interpretability and reducing input dimensionality, model complexity can be minimized, enabling on-device inference where data remain local. In such privacy-preserving architectures, models could be deployed in a federated manner—trained centrally but executed locally—with users retaining control over whether to share anonymized data for model improvement. Second, clinical integration may follow a phased roadmap: initial testing within research centres, scaling through larger volunteer-based deployments, and eventual embedding into clinical dashboards that complement consultations. These dashboards could provide intermediate estimates or alerts when deviations exceed expected individual fluctuations. Third, minimizing risks of over-detection or false reassurance will depend on incremental validation in progressively more diverse populations, together with the development of models that improve both sensitivity and specificity.

Future work should further detail the predictive feature contributions for each of the outcomes and investigate the implications of these modality-specific patterns for personalized and adaptive digital brain health monitoring systems. Models combining all the outcomes should also be explored to account for interactions between them (e.g., stress affecting memory performance).

In conclusion, this study presents an explainable framework for continuous, passive monitoring of everyday brain health using consumer-grade mobile and wearable data. By integrating behavioural, physiological, and environmental signals, we predicted multiple cognitive and affective outcomes with low error rates. Beyond predictive accuracy, feature-importance analyses revealed complementary roles of ambient exposures and physiological rhythms—where environmental factors better explained stable inter-individual differences, while behavioural and circadian patterns captured within-person fluctuations over time. These findings demonstrate that real-world passive data can capture meaningful dynamics in cognition and affect, supporting scalable and low-burden approaches for population-level brain-health monitoring. Rather than replacing clinical assessment, such methods lay the groundwork for proactive, data-driven strategies to monitor mental well-being and detect early deviations in cognitive or affective trajectories. Future research should validate these models across longer time spans and diverse populations to confirm their robustness and clinical utility.

## Methods

### Data Collection

This research is part of the *Providemus alz* project^26^ conducted at the University of Geneva, Switzerland. It is a longitudinal observational study in cognitively healthy volunteers, with data collection starting in March 2024 and planned to end by March 2026. Eligibility for participation was defined by the inclusion criteria of (a) being 45 years or older, (b) residing in Switzerland or surrounding French regions, (c) being fluent in English, French, or Portuguese, (d) using a smartphone daily, and (e) being able to wear and charge a provided smartwatch. Participants were excluded if reporting any diagnosis of mental health issues. Ethical clearance was granted by the *Commission Cantonale d’Éthique de la Recherche sur l’être humain* with the number 2023-00975, and all participants provided written consent before enrolment.

The data used in this article refers to a sample of 82 participants of that study, with their baseline characteristics provided in Table 6.

**Table 6:** Baseline characteristics

	Cognitively healthy (n = 82)
Sex at birth	
Female	49 (59.80%)
Male	33 (40.20%)
Age at enrolment (years)	
Mean (SD)	57.70 (8.49)
Range	45.54–77.62
Ethnic origin	
White	76 (92.68%)
Asian	2 (2.44%)
Hispanic Latino or Spanish	2 (2.44%)
Other	2 (2.44%)
Language used during the study	
French	61 (74.40%)
English	20 (24.40%)
Portuguese	1 (1.20%)
Native language used during the study	
Yes	61 (74.40%)
No	21 (25.60%)
Education (years, reported across waves)	
Mean (SD)	17.83 (4.71)
Range	6 - 37.70
Dementia cases in the family	
No	52 (63.40%)
Yes	27 (32.90%)
Don’t know	3 (3.70%)

Collection of active data implied a direct input from the participant and could be a *Patient-Reported Outcome* (PRO), which refers to validated questionnaires self-administered, or a *Performance-Reported Outcome* (PerfRO), which denotes cognitive performance tasks. Passive data, *Technology-Reported Outcomes* (TechRO), was collected without need for participants’ action, which link to metrics obtained passively using digital sensors. A comprehensive list is presented in Table 7, with units’ description in Table S9 of the Supplementary Materials. An earlier publication on the protocol^26^ includes further details.

**Table 7:** Listing of active and passive data collected

Active data outcomes per wave (21)	Passive data metrics(40, all TechROs)
PROs (8, 38% of 21): - Anxiety (AState) - Cognitive decline (Cogn) - Depression (AState) - Hostility (AState) - Memory complaints (Cogn) - Negative affect (AState) - Positive affect (AState) - Stress (AState) PerfROs (13, 62%): - Attention (Cogn) - Cognitive flexibility (Cogn) - Inductive reasoning (Cogn) - Inhibitory control (Cogn) - Long-term memory (Cogn) - Processing speed (Cogn) - Prospective memory (Cogn) - Short-term memory (Cogn) - Tapping speed (Cogn) - Typing speed (Cogn) - Verbal fluency (2 versions) (Cogn) - Working memory (Cogn)	Physical activity (5, 12.5% of 40): - Active calories burned (T) - Distance walking or running (T) - Distance rate (M) - Steps (T) - Step frequency (M) Sleep (10, 25%): - Deep sleep duration (T) - Light sleep duration (T) - Sleep efficiency - Sleep latency - Sleep score - Time spent in bed (T) - Total sleep time - Wake-up count (T) - Wake-up latency - *Wakefulness after sleep onset* (WASO) Sleep HR (3, 7.5%): - Sleeping HR (M, Min, Max) 24 h HR (5, 12.5%): - 24-h HR (M, SD, Mdn, Min, Max) Weather (6, 15%): - Air temperature - Atmospheric pressure - Humidity - Maximum air temperature forecasted - Minimum air temperature forecasted - Temperature feeling Atmospheric pollutants (9, 22.5%): - *Air quality index* (AQI) - Ammonia (NH_3_) - Carbon monoxide (CO) - Ozone (O_3_) - Nitric oxide (NO) - Nitrogen dioxide (NO_2_) - *Particulate matter 2.5* (PM 2.5) - *Particulate matter 10* (PM 10) - Sulfur dioxide (SO_2_) Other (2, 5%): - Time zone difference (day to day) - Wear time day percentage (T)

Units are specified in Table S9 in the Supplementary Materials.

Active data included validated questionnaires and tests able to assess *cognition* (Cogn) and *affective state* (AState). A total of 21 active data outcomes (i.e., target variables or output features) were measured at every one of four waves. These were collected using the Buss-Durkee Hostility Inventory^29^ (hostility), the Cognitive Telephone Screening Instrument^30^ (inductive reasoning, long-term, prospective, short-term, and working memories, typing speed, and verbal fluency), the Hospital Anxiety and Depression Scale^31^ (anxiety and depression), the Informant Questionnaire on Cognitive Decline in the Elderly^32^ (cognitive decline), the Positive and Negative Affect Scale^33^ (PANAS, positive and negative affect), the Prospective and Retrospective Memory Questionnaire^34^ (memory complaints), the Perceived Stress Scale^35^ (stress), and the finger-tapping^36^ (tapping speed), flanker^37^ (inhibitory control), reaction time^38^ (attention), and trail making^39^ (cognitive flexibility, processing speed) tests. For clarity, positive and negative affect correspond to the two core dimensions of the PANAS: positive affect reflects active, pleasurable emotional states (e.g., enthusiasm, alertness), while negative affect reflects distress-related states (e.g., nervousness, irritability); each is computed as the sum of its respective items. Additionally, serving as controls in the modelling phase, we included PROs collected (a) only at baseline, like Body Mass Index at the age of 40, sex, scores of the *Cognitive Reserve Index Questionnaire*^40^ (CRIQ) and of the *Mediterranean Diet Score*^41^ (MDS), and (b) at the start of every wave, like smoking and alcohol consumption, cardiovascular or sleep disorders diagnosis, occurrences of head injuries, and participants‘ subjective age.

Forty passive data types (i.e., input features) were used, consisting of physiological and behavioural factors previously identified as potentially linked to changes in everyday brain health, as well as environmental and behavioural variables; these were grouped into seven distinct passive data metric groups (Table 7).

A custom-designed smartphone application *mQoL* (*mQoL Living Lab infrastructure*^42^) was installed on the participant’s personal smartphone to collect all the PROs, PerfROs, and the environmental TechROs. For the remaining TechROs, a hybrid smartwatch *Withings Steel HR* was used to provide data on heart rate^43^, physical activity^44,45^, and sleep^44^ with previously tested accuracy. In the research presented here, we use this data in the format of daily summaries (i.e., aggregating all data points within a day) using eight descriptive statistics: *mean* (M), *standard deviation* (SD), *median* (Mdn), *minimum value* (Min), *maximum value* (Max), and *daily total* (T).

**Fig. 7:**
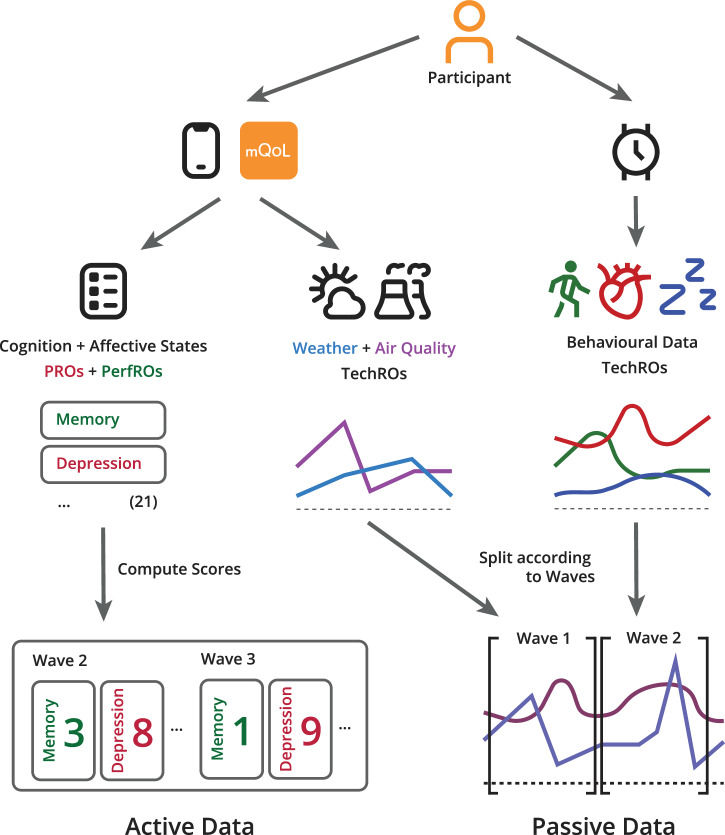
Data collection and alignment of active and passive measures. Schematic overview of data collected via the mQoL mobile application (left) and the smartwatch (right), their transformation, and combination into the active and passive data used in modelling.

The different data sources and types are depicted in Fig. 7. For clarity, some are described below:

- Distance rate: computed by dividing the distance covered in a day by the total wear time in seconds. It informs us how much distance is covered in each second of wear time.
- Sleep score: value ranging between 0 (worst) and 100 (best), computed by the smartwatch's manufacturer's proprietary algorithms.
- Step frequency: computed by dividing the total number of steps taken in a day by the total wear time. It informs us how frequent walking or running was.

To better interpret the proprietary Sleep Score metric, we examined its relationship with other wearable-derived sleep variables. Correlations were computed using Pearson’s, Spearman’s, and Kendall’s coefficients across all participants concatenated daily data. The Sleep Score showed the strongest positive correlations with total sleep time (*r* = 0.72, 0.76, 0.60), time in bed (*r* = 0.66, 0.69, 0.52), and deep sleep duration (*r* = 0.63, 0.71, 0.52), and weaker or negligible correlations with heart-rate metrics and wakefulness indicators, suggesting that it reflects a composite of sleep quantity and quality (see Table S1 in the Supplementary Materials).

### Study procedure

**Fig. 8:**
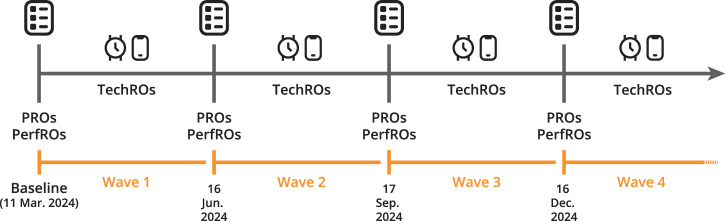
Study timeline and assessment waves. Overview of the longitudinal study design from the March 2024 baseline and four assessment waves conducted approximately every three months. Active assessments were collected at the start of each wave, while passive data were continuously collected throughout the study.

Figure 8 illustrates the timeline of the *Providemus alz* study, which will comprise nine waves in total. In this study, a wave refers to a new assessment round of the same participant cohort, not a new group of participants. Each wave therefore captures repeated active measurements (PROs and PerfROs) while passive data collection continues continuously in between. At each wave’s start, all participants were informed by push notifications within the mQoL app and by an email about the new set of tasks to complete and were instructed to complete all of those within 14 days. This time window was chosen based on literature from digital health adherence research showing that extending availability beyond one to two weeks improves completion rates while minimizing perceived burden and participant fatigue in longitudinal studies^46,47^. However, participants could choose to complete these upon their own will—it was possible to split the tasks across several days, choose the order, vary the moment of the day, complete tasks outside the 14-day window or even skip a wave. The three-month interval between waves was selected as a balance between temporal sensitivity, practice effects, and burden feasibility^46,48^. A shorter interval could have increased response fatigue and redundancy across waves, whereas a longer interval would reduce temporal resolution and the ability to model within-person changes. The quarterly schedule thus represents a feasible compromise for long-term adherence and comparability with existing cognitive-affective longitudinal studies^46,48^.

### Data quality control

Following the literature^49,50^, a moment of non-wear time was defined as at least 60 consecutive minutes with zero readings from all sensors. We recorded these moments as missing data. A day of data was considered valid for analysis if containing at least 10 h of wear time. Considering the duration of each wave, a participant’s data was validated for analysis if having at least 50% of the wave duration as valid days. Regarding atmospheric data, there was a minimum of one data point of weather and air quality for each wave per participant. No data imputation was performed on the predictors or outcomes. Additionally, a minimum of one step per day of data and a minimum of 20 for HR mean measurements a day were required to be consider that data valid. We applied these thresholds to identify and discard potential sensor failures. Because the data were aggregated daily and required at least 10 h of wear time, a zero-step count would not represent a realistic scenario, and daily HR means below 20 bpm fall below the physiological lower limit observed even in the most active individuals^51^.

### Data representations and feature extraction

Three different ways of representing data (both active and passive) along the waves were studied, defined for each participant as:

1. *Levels*: observed outcome value. Active data values were to be predicted by passive data values preceding the assessment.
2. *Absolute drifts* (ΔABS): value at a given wave minus the value at a preceding wave. Each participant therefore had three pairs of ΔABS corresponding to three pairs of consecutive waves.
3. *Proportional drift* (Δ%): difference of values as a percentage of the value in the preceding wave.

Additionally, *subjective age difference* was computed as the subtraction of the indicated subjective age’s midpoint from the chronological age at the time of assessment, and *time difference* was computed to represent the time between two consecutive active assessments for all the ΔABS and Δ%.

Each wave’s passive data was represented by eight summary features (i.e., descriptive statistics): M, SD, Mdn, *interquartile range* (IQR), Min, Max, s*kewness* (Skew), and *kurtosis* (Kurt). An illustration of the data representations, features extracted, and data merging can be found in the Supplementary Materials (Fig. S1).

### Feature selection

Before modelling, we performed feature selection to avoid duplicated information and overfit, by using Pearson, Spearman, and Kendall partial correlations for each combination of the 21 outcomes and 40 passive data metrics. Four additional control variables were added as predictors; hence, the correlations were partial rather than univariate. These control variables were chronological age and subjective age difference (both computed for each wave), sex, and the education score of CRIQ (both at baseline). To reduce the impact of outliers at both ends of the correlation analysis and prioritize method replication, we implemented Z-score normalization.

For each of the 21 active outcomes, we proceeded as follows. For each type of correlation, we selected the daily summary feature (out of eight) of each predictor with the biggest absolute correlation (magnitude/absolute value). This resulted in 40 selected features per type of correlation, one for each predictor; see Fig. S2 in the Supplementary Material. We thereby examined linear relationships (Pearson) as well as non-linear but monotonic associations (Spearman, Kendall).

### Modelling

Although most of the active outcomes were assessed as psychometrically validated discrete ordinal variables, we analyzed them as continuous variables to account for the transition between discrete values. This is reasonable given our study objective to elaborate on a continuous measurement of changes that happen in a continuous way. Thus, we employed Artificial Intelligence using three decision tree-based algorithms, and one non-tree based: *Random Forest* (RF), *Light Gradient Boosting Machine* (LightGBM), and *eXtreme Gradient Boosting* (XGBoost), and *Support Vector Machine* (SVM). We selected such models because they perform reliably with small-to-moderate sample sizes, nonlinear relationships, and heterogeneous feature distributions, and they offer interpretable feature importance; deep learning methods were avoided due to sample size constraints. To directly compare the results of all models, we applied Z-score normalization to the features and the outcome of all models before fitting.

The first round of modelling served the purpose of filtering the most important features for each outcome by applying two feature importance explanation techniques: *Shapley-based explanations* (SHAP) for RF, LightGBM, and XGBoost, and *Permutation Feature Importance* (PFI) for SVM (due to technical limitations making SHAP unusable for SVM).

When filtering the top features for the second round, we did it per model per outcome. From the tree-based models, we selected the group of input variables with a cumulative SHAP of at least 0.90, after having ordered the features according to the absolute SHAP value (in decreasing order). For SVM models we filtered the features with a positive PFI score.

After filtering the top features for each outcome model, all were retrained and reevaluated. To evaluate the model’s accuracy when predicting each of the outcomes, we performed two types of *Cross Validation* (CV): user-dependent (up to 82 folds) and wave-dependent (up to four folds). The two cross-validation schemes were designed to test distinct aspects of generalization. The user-dependent CV (subject-independent) evaluates how well the models generalize across individuals—i.e., whether the learned relationships between passive and active data transfer to new participants not seen during training. In contrast, the wave-dependent CV (time-dependent) assesses temporal generalization, testing the model’s ability to predict new waves of data from the same individuals while accounting for potential seasonal or temporal fluctuations in everyday life. Together, these two approaches capture both between-person and within-person predictive robustness, providing insight into the applicability of the models. In the wave-dependent CV, folds were defined strictly by wave index, ensuring that the model was trained on all but one wave and tested on the held-out wave. Passive input features were always restricted to the period preceding each wave’s active assessments, so that no future information was used to predict earlier outcomes. This setup isolates temporal generalization effects without introducing information leakage^52^.

In each step of each CV, the model was evaluated using *Mean Absolute Error* (MAE). That MAE was then converted to reflect the outcome’s range “scaled error”; that is, we divided each MAE value by the literature-based range of values for the respective active data type. We hereby refer to this metric as *Scaled MAE* (SMAE). For outcomes with clearly validated theoretical ranges (13), we retained the literature-defined min–max values to avoid overestimating performance and to preserve consistency with the psychometric tools’ original scaling conventions. Eight other outcomes (all PerfROs) did not have literature-defined ranges available. For seven of those, empirical ranges were defined as *M* + *2* *SD—lowest observed value* to represent around 95% coverage. For one remaining outcome, whose values spanned both negative and positive domains, we defined the range symmetrically using ± 2 SD bounds—that is, *M* + *2* *SD of the positive values minus M* − *2* *SD of the negative values*—ensuring comparable coverage. The choice of ±2 SD was made to account for real-life noise such as device issues (e.g., touch screen irresponsiveness due to software issues), momentary user inattention, or misunderstanding of task instructions. Such irregularities when assessing PerfROs can produce extreme values that do not meaningfully reflect the underlying cognitive or affective construct being assessed. Using min–max scaling in this context would anchor the model evaluation to these atypical extremes, artificially inflating the permissible error and obscuring the model’s true predictive capability. By contrast, a ± 2 SD interval allows the SMAE to be calibrated to the typical empirical variation in real-world performance, while down-weighting rare, non-informative, or erroneous observations—an unavoidable aspect of remote, unsupervised cognitive assessment. Table S2 in the Supplementary Materials details the size of the interval of values used; Figs. S3-S4 depict the second modelling phase.

## Supplementary information

Supplementary Information

## Data Availability

Deidentified participant data underlying the findings of this study, along with the data dictionary, will be made available upon reasonable request to the first author. Access to the raw data is restricted due to ethical and privacy constraints and will require a data access agreement. Access to the raw data may be granted to researchers whose proposed use of the data has been approved by the study team.

## References

[1] Gamaldo, A. A. & Allaire, J. C. Daily fluctuations in everyday cognition: is it meaningful? *J. Aging Health* **28**, 834–849 (2016). 26538267 10.1177/0898264315611669

[2] Hanushek, E. A., Kinne, L., Witthöft, F. & Woessmann, L. Age and cognitive skills: use it or lose it. *Sci. Adv.* **11** (2025). 10.1126/sciadv.ads1560 PMC11881919 40043134

[3] Brain health. https://www.who.int/health-topics/brain-health#tab=tab_1.

[4] Gamaldo, A. A. & Allaire, J. C. Daily fluctuations in everyday cognition. *J. Aging Health* **28**, 834–849 (2015). 26538267 10.1177/0898264315611669

[5] Brasier, N. et al. Next-generation wearable sensors for biopsychosocial care in mental health: a narrative review. *BMJ Digital Health AI* **1**, e000018 (2025). 10.1136/bmjdhai-2025-000018 PMC13492488 42712275

[6] Chen, R. et al. Developing measures of cognitive impairment in the real world from consumer-grade multimodal sensor streams. In *Proc. ACM SIGKDD International Conference on Knowledge Discovery and Data Mining* 11, 2145–2155 (2019).

[7] Sliwinski, M. J. et al. Reliability and validity of ambulatory cognitive assessments. *Assessment* **25**, 14 (2016). 27084835 10.1177/1073191116643164 PMC5690878

[8] Powell, D. et al. Exploring the potential of digital biomarkers as a measure of brain health ‘capital. *NPJ Digit Med.* **8**, 1–4 (2025). 40473915 10.1038/s41746-025-01675-2 PMC12141678

[9] Kourtis, L. C., Regele, O. B., Wright, J. M. & Jones, G. B. Digital biomarkers for Alzheimer’s disease: the mobile/wearable devices opportunity. *NPJ Digit Med.* **2**, 1–9 (2019). 10.1038/s41746-019-0084-2 PMC6526279 31119198

[10] Baglioni, C. et al. Interactions between insomnia, sleep duration and emotional processes: an ecological momentary assessment of longitudinal influences combining self-report and physiological measures. *J. Sleep. Res.* **33**, e14001 (2024). 37491710 10.1111/jsr.14001

[11] Beattie, L., Kyle, S. D., Espie, C. A. & Biello, S. M. Social interactions, emotion and sleep: a systematic review and research agenda. *Sleep. Med. Rev.* **24**, 83–100 (2015). 25697832 10.1016/j.smrv.2014.12.005

[12] Magnon, V. et al. Does heart rate variability predict better executive functioning? A systematic review and meta-analysis. *Cortex* **155**, 218–236 (2022). 36030561 10.1016/j.cortex.2022.07.008

[13] Keller, M. C. et al. A warm heart and a clear head. *Psychol. Sci.* **16**, 724–731 (2005). 16137259 10.1111/j.1467-9280.2005.01602.x

[14] Taylor, L., Watkins, S. L., Marshall, H., Dascombe, B. J. & Foster, J. The impact of different environmental conditions on cognitive function: a focused review. *Front Physiol.* **6**, 372 (2016). 26779029 10.3389/fphys.2015.00372 PMC4701920

[15] Yang, T., Wang, J., Huang, J., Kelly, F. J. & Li, G. Long-term exposure to multiple ambient air pollutants and association with incident depression and anxiety. *JAMA Psychiatry* **80**, 305–313 (2023). 36723924 10.1001/jamapsychiatry.2022.4812 PMC10077109

[16] Bhui, K. et al. Air quality and mental health: evidence, challenges and future directions. *BJPsych Open* **9**, e120 (2023). 37403494 10.1192/bjo.2023.507 PMC10375903

[17] Van Der Roest, H. G. et al. Subjective needs of people with dementia: a review of the literature. *Int. Psychogeriatr.* **19**, 559–592 (2007). 17201993 10.1017/S1041610206004716

[18] Ford, E., Milne, R. & Curlewis, K. Ethical issues when using digital biomarkers and artificial intelligence for the early detection of dementia. *Wiley Interdiscip Rev Data Min Knowl Discov.* **13** (2023). 10.1002/widm.1492 PMC10909482 38439952

[19] Juul Rasmussen, I. & Frikke-Schmidt, R. Modifiable cardiovascular risk factors and genetics for targeted prevention of dementia. *Eur. Heart J.* **44**, 2526–2543 (2023). 37224508 10.1093/eurheartj/ehad293 PMC10481783

[20] Butler, P. M. et al. Smartwatch- and smartphone-based remote assessment of brain health and detection of mild cognitive impairment. *Nat. Med.* 1–11. (2025). 10.1038/s41591-024-03475-9 PMC11922773 40038507

[21] Iulita, M. F., Streel, E. & Harrison, J. Digital biomarkers: Redefining clinical outcomes and the concept of meaningful change. *Alzheimer’s. Dement.: Transl. Res. Clin. Interventions* **11**, e70114 (2025). 10.1002/trc2.70114 PMC12130567 40463636

[22] Zhou, H. et al. Digital biomarkers of cognitive frailty: the value of detailed gait assessment beyond gait speed. *Gerontology* **68**, 224–233 (2022). 33971647 10.1159/000515939 PMC8578566

[23] Exler, A., Klebsattel, C., Schankin, A. & Beigl, M. A wearable system for mood assessment considering smartphone features and data from mobile ECGs. *UbiComp 2016 Adjunct**—Proceedings of t**he 2016 ACM International Joint Conference on Pervasive and Ubiquitous Computing,* 1153–1161 (2016).

[24] Cormack, F. et al. Wearable technology for high-frequency cognitive and mood assessment in major depressive disorder: Longitudinal observational study. *JMIR Ment. Health* **6**, e12814 (2019). 31738172 10.2196/12814 PMC6887827

[25] Hickman, R., D’Oliveira, T. C., Davies, A. & Shergill, S. Monitoring daily sleep, mood, and affect using digital technologies and wearables: a systematic review. *Sensors* **24**, 4701 (2024). 39066098 10.3390/s24144701 PMC11280943

[26] Matias, I., Kliegel, M. & Wac, K. Providemus alz: Ubiquitous Screening of Preclinical Alzheimer's Disease with Consumer-grade Technologies. In *UbiComp Companion 2024**—Companion of the 2024 ACM International Joint Conference on Pervasive and Ubiquitous Computing,* 743–751 (Association for Computing Machinery, Inc, 2024).

[27] Henrich, J., Heine, S. J. & Norenzayan, A. The weirdest people in the world? *Behav. Brain Sci.* **33**, 61–83 (2010). 20550733 10.1017/S0140525X0999152X

[28] Walls, T. A. & Schafer, J. L. *Models for Intensive Longitudinal Data*. *Models for Intensive Longitudinal Data* (Oxford University Press, 2012).

[29] Buss, A. H. & Durkee, A. An inventory for assessing different kinds of hostility. *J. Consult Psychol.* **21**, 343–349 (1957). 13463189 10.1037/h0046900

[30] Haas, M., Scheibe, S. & El Khawli, E. Online assessment of cognitive functioning across the adult lifespan using the eCOGTEL: a reliable alternative to laboratory testing. *Eur. J. Ageing*. **19**, 609–619 (2022). 10.1007/s10433-021-00667-x PMC8655327 34903960

[31] Zigmond, A. S. & Snaith, R. P. The hospital anxiety and depression scale. *Acta Psychiatr. Scand.* **67**, 361–370 (1983). 6880820 10.1111/j.1600-0447.1983.tb09716.x

[32] Jorm, A. F. & Jacomb, P. A. The Informant Questionnaire on Cognitive Decline in the Elderly (IQCODE): Socio-demographic correlates, reliability, validity and some norms. *Psychol. Med* **19**, 1015–1022 (1989). 2594878 10.1017/s0033291700005742

[33] Watson, D., Clark, L. A. & Tellegen, A. Development and validation of brief measures of positive and negative affect: the PANAS scales. *J. Pers. Soc. Psychol.* **54**, 1063–1070 (1988). 3397865 10.1037//0022-3514.54.6.1063

[34] Smith, G., Della Sala, S., Logie, R. H. & Maylor, E. A. Prospective and retrospective memory in normal ageing and dementia: a questionnaire study. *Memory* **8**, 311–321 (2000). 11045239 10.1080/09658210050117735

[35] Cohen, S., Kamarck, T. & Mermelstein, R. A global measure of perceived stress. *J. Health Soc. Behav.* **24**, 385–396 (1983). 6668417

[36] Ward, T. et al. Finger-Tapping Test. In *Encyclopedia of Autism Spectrum Disorders,* 1296–1296 (Springer, 2013).

[37] Eriksen, B. A. & Eriksen, C. W. Effects of noise letters upon the identification of a target letter in a nonsearch task. *Percept. Psychophys.* **16**, 143–149 (1974).

[38] Deary, I. J., Liewald, D. & Nissan, J. A free, easy-to-use, computer-based simple and four-choice reaction time programme: The Deary-Liewald reaction time task. *Behav. Res Methods* **43**, 258–268 (2011). 21287123 10.3758/s13428-010-0024-1

[39] Tombaugh, T. N. Trail making test A and B: normative data stratified by age and education. *Arch. Clin. Neuropsychol.* **19**, 203–214 (2004). 15010086 10.1016/S0887-6177(03)00039-8

[40] Nucci, M., Mapelli, D. & Mondini, S. Cognitive Reserve Index questionnaire (CRIq): a new instrument for measuring cognitive reserve. *Aging Clin. Exp. Res* **24**, 218–226 (2012). 21691143 10.3275/7800

[41] Panagiotakos, D. B., Pitsavos, C. & Stefanadis, C. Dietary patterns: A Mediterranean diet score and its relation to clinical and biological markers of cardiovascular disease risk. *Nutr., Metab. Cardiovasc. Dis.* **16**, 559–568 (2006). 17126772 10.1016/j.numecd.2005.08.006

[42] Berrocal, A., Manea, V., de Masi, A. & Wac, K. MQOL lab: Step-by-step creation of a flexible platform to conduct studies using interactive, mobile, wearable and ubiquitous devices. In *Procedia Computer Science* Vol. 175, 221–229 (Elsevier, 2020).

[43] Helmer, P. et al. Accuracy and systematic biases of heart rate measurements by consumer-grade fitness trackers in postoperative patients: prospective clinical trial. *J. Med. Internet Res.* **24** (2022). 10.2196/42359 PMC9840097 36583938

[44] Ferguson, T., Rowlands, A. V., Olds, T. & Maher, C. The validity of consumer-level, activity monitors in healthy adults worn in free-living conditions: a cross-sectional study. *Int. J. Behav. Nutr. Phys. Act* **12** (2015). 10.1186/s12966-015-0201-9 PMC4416251 25890168

[45] An, H. S., Jones, G. C., Kang, S. K., Welk, G. J. & Lee, J. M. How valid are wearable physical activity trackers for measuring steps? *Eur. J. Sport Sci.* **17**, 360–368 (2017). 27912681 10.1080/17461391.2016.1255261

[46] Bartels, C., Wegrzyn, M., Wiedl, A., Ackermann, V. & Ehrenreich, H. Practice effects in healthy adults: A longitudinal study on frequent repetitive cognitive testing. *BMC Neurosci.* **11**, 118 (2010). 20846444 10.1186/1471-2202-11-118 PMC2955045

[47] Butler, P. M. et al. Smartwatch- and smartphone-based remote assessment of brain health and detection of mild cognitive impairment. *Nat. Med.* **31**, 829–839 (2025). 40038507 10.1038/s41591-024-03475-9 PMC11922773

[48] Salthouse, T. A. Frequent assessments may obscure cognitive decline. *Psychol. Assess.* **26**, 1063 (2014). 24840179 10.1037/pas0000007 PMC4237696

[49] Tudor-Locke, C., Camhi, S. M. & Troiano, R. P. A catalog of rules, variables, and definitions applied to accelerometer data in the national health and nutrition examination Survey, 2003-2006. *Prev. Chronic Dis.* **9** (2012). 10.5888/pcd9.110332 PMC3457743 22698174

[50] Di, J. et al. Considerations to address missing data when deriving clinical trial endpoints from digital health technologies. *Contemp. Clin. Trials* **113** (2022). 10.1016/j.cct.2021.106661 34954098

[51] Speed, C. et al. Measure by measure: Resting heart rate across the 24-hour cycle. *PLOS Digital Health* **2**, e0000236 (2023). 37115739 10.1371/journal.pdig.0000236 PMC10146540

[52] Kapoor, S. & Narayanan, A. Leakage and the reproducibility crisis in machine-learning-based science. *Patterns* **4**, 100804 (2023). 37720327 10.1016/j.patter.2023.100804 PMC10499856

