# Author Correction: Digital biomarkers for brain health: passive and continuous assessment from wearable sensors

**DOI:** 10.1038/s41746-026-03031-4

**Published:** 2026-08-01

**Authors:** Igor Matias, Maximilian Haas, Eric J. Daza, Matthias Kliegel, Katarzyna Wac

**Affiliations:** 1 Quality of Life Technologies Lab, University of Geneva, Geneva, Switzerland; 2 Cognitive Aging Lab, University of Geneva, Geneva, Switzerland; 3 Faculty of Psychology, UniDistance Suisse, Geneva, Switzerland; 4 Stats-of-1, Menlo Park, CA USA; 5 Boehringer Ingelheim Pharmaceuticals Inc., Ridgefield, CA USA

**Keywords:** Biomarkers, Health care, Neuroscience

Correction to: *npj Digital Medicine* 10.1038/s41746-026-02340-y, published online 14 January 2026

In the previous version, the “Male” and “Female” labels in Table 6 of the published article were inadvertently swapped. As a result, the table currently indicates that approximately 60% of the participants were male, whereas the correct value is approximately 60% female.

