# Supplementary material for: Digital biomarkers for brain health: passive and continuous assessment from wearable sensors
Source: NPJ Digit Med. 2026 Jan 14;9:197. doi: 10.1038/s41746-026-02340-y (PMC12957369; doi:10.1038/s41746-026-02340-y)
Supplement: Supplementary file 1 — Supplementary Information [file 41746_2026_2340_MOESM1_ESM.pdf]

Supplementary Materials for

Digital Biomarkers for Brain Health: Passive and Continuous Assessment from Wearable Sensors

Igor Matias*, Maximilian Haas, Eric J. Daza, Matthias Kliegel, Katarzyna Wac

*corresponding author: igor.matias@unige.ch

Supplementary Material A: Supplementary Tables

Table S1 – Correlations between the proprietary Sleep Score and other wearable-derived sleep features using Pearson's r, Spearman's ρ, and Kendall's τ coefficients.

Table S2 – Range of values used while computing the SMAE for all the 21 active data outcomes.

Table S3 – Ratio of features selected for the first round of modelling of passive data.

Table S4 – Number of samples used for modelling each active data outcome after standardization and removal of missing data points.

Table S5 – Comparison of prediction errors between the population mean predictor (naïve model) and the machine learning model achieving the lowest SMAE.

Table S6 – Mean and SD overlap coefficients quantifying the proportion of features selected in the first modelling round that would have remained selected if each participant was excluded per fold (leave-one-out analysis).

Table S7 – Mean and SD Jaccard indices comparing the set of features selected in the first modelling round that would have remained selected if each participant was excluded per fold (leave-one-out analysis).

Table S8 – Top three predictors contributing most to the prediction of each cognitive and affective outcome, based on mean feature importance values from the model with the lowest SMAE under user-dependent cross-validation.

Table S9 – Units of the passive data metrics collected.

Supplementary Material B: Supplementary Figures

Figure S1 – Illustration of the different data representations, features extracted, and final merging of active with passive data.

Figure S2 – Illustration of the feature selection process applied to each data representation and each correlation coefficient.

Figure S3 – Illustration of the steps undertaken for the first stage of modelling.

Figure S4 – Illustration of the steps between the first round of modelling and the final results of our research.

Figure S5 – Feature importance across groups of passive metrics for the most accurate models according to the Mean SMAE using user-dependent CV.

Figure S6 – Feature importance across groups of passive metrics for the most accurate models according to the Mean SMAE using wave-dependent CV.

Figure S7 – Feature importance across groups of passive metrics for the most accurate models according to the Median SMAE using user-dependent CV.

Figure S8 – Feature importance across groups of passive metrics for the most accurate models according to the Median SMAE using wave-dependent CV.

Supplementary Material C: Other Supplementary Materials

Supplementary Material C 1: Description of data used after data exclusion.

Supplementary Material C 2: Descriptive Analysis of the Metrics Computed.

Supplementary Material C 3: Temporal Dynamics of the Used Data.

Supplementary Material A: Supplementary Tables

Table S1 – Correlations between the proprietary Sleep Score and other wearable-derived sleep features using Pearson's r, Spearman's ρ, and Kendall's τ coefficients. Each cell reports the correlation coefficient (and p-value) based on all concatenated participant data (Levels representation). Units are specified in Table S9.

Passive Data Metric	Pearson r (p-value)	Spearman r (p-value)	Kendall r (p-value)
Deep sleep duration	+ 0.625 (0.000)	+ 0.713 (0.000)	+ 0.524 (0.000)
Light sleep duration	+ 0.384 (0.000)	+ 0.290 (0.000)	+ 0.192 (0.000)
Sleep efficiency	+ 0.280 (0.000)	+ 0.341 (0.000)	+ 0.250 (0.000)
Sleep latency	- 0.026 (0.000)	+ 0.018 (0.013)	+ 0.014 (0.013)
Time spent in bed	+ 0.661 (0.000)	+ 0.686 (0.000)	+ 0.520 (0.000)
Total sleep time	+ 0.722 (0.000)	+ 0.761 (0.000)	+ 0.602 (0.000)
Wake-up count	- 0.017 (0.015)	- 0.124 (4.31×10^{-69})	- 0.095 (5.25×10^{-75})
Wake-up latency	- 0.004 (0.552)	+ 0.008 (0.251)	+ 0.006 (0.248)
WASO	- 0.025 (0.001)	- 0.148 (4.99×10^{-98})	- 0.107 (3.55×10^{-107})
Sleeping HR mean	- 0.022 (0.002)	- 0.061 (1.04×10^{-17})	- 0.042 (1.08×10^{-17})
Sleeping HR minimum	+ 0.046 (9.56×10^{-11})	- 0.001 (0.931)	- 0.001 (0.896)
Sleeping HR maximum	- 0.049 (3.63×10^{-12})	- 0.079 (7.17×10^{-29})	- 0.054 (1.12×10^{-28})

*Table S2 – Range of values used while computing the SMAE for all the 21 active data outcomes. PerfROs are marked with *.*

Active data type (ref)	Range of values	Calculation method
Anxiety [1]	22	Literature-defined
Cognitive decline [2]	5	Literature-defined
Depression [1]	22	Literature-defined
Hostility [3]	4	Literature-defined
Memory complaints [4]	65	Literature-defined
Negative affect [5]	41	Literature-defined
Positive affect [5]	41	Literature-defined
Stress [6]	41	Literature-defined
Attention * [7]	3203.73	M + 2 SD – lowest observed value
Cognitive flexibility * [8]	25.24	M + 2 SD – lowest observed value
Inductive reasoning * [9]	9	Literature-defined
Inhibitory control * [10]	2260.68	Pos. (M + 2 SD) – Neg. (M - 2 SD)
Long-term memory * [9]	9	Literature-defined
Processing speed * [8]	12.34	M + 2 SD – lowest observed value
Prospective memory * [9]	2	Literature-defined
Short-term memory * [9]	9	Literature-defined
Tapping speed * [11]	424.52	M + 2 SD – lowest observed value
Typing speed * [9]	2.63	M + 2 SD – lowest observed value
Verbal fluency exercise 1 * [9]	18.89	M + 2 SD – lowest observed value
Verbal fluency exercise 2 * [9]	16.42	M + 2 SD – lowest observed value
Working memory * [9]	13	Literature-defined

Table S3 - Ratio of features selected for the first round of modelling of passive data.

**Levels**			**Absolute drifts (ΔABS)**			**Proportional drifts (Δ%)**		
**Pearson (%)**	**Spearman (%)**	**Kendall (%)**	**Pearson (%)**	**Spearman (%)**	**Kendall (%)**	**Pearson (%)**	**Spearman (%)**	**Kendall (%)**
Kurt (20.1)	Kurt (20.9)	Min (20.7)	Kurt (16.5)	IQR (17.8)	IQR (17.5)	Min (22.7)	Min (22.5)	Min (23.3)
Min (18.8)	Min (19.3)	Kurt (20.0)	Min (16.3)	Min (16.1)	Min (17.4)	Kurt (19.6)	Kurt (21.7)	Kurt (22.0)
Skew (14.5)	IQR (13.2)	IQR (13.1)	IQR (15.2)	Kurt (15.3)	Kurt (15.3)	Skew (13.3)	Skew (13.0)	Skew (13.6)
IQR (13.6)	Skew (12.7)	Skew (12.7)	Skew (12.3)	Mdn (14.2)	Mdn (14.4)	Mdn (12.3)	Mdn (11.9)	IQR (10.3)
Mdn (12.6)	Mdn (12.1)	Mdn (11.0)	SD (11.9)	Skew (12.3)	SD (11.6)	IQR (10.0)	IQR (10.8)	Mdn (10.3)
SD (10.3)	SD (10.8)	SD (10.5)	Mdn (11.7)	SD (11.9)	Skew (11.4)	SD (7.9)	SD (7.9)	M (8.2)
M (5.8)	M (6.5)	M (6.9)	Max (11.0)	Max (7.8)	Max (7.8)	Max (7.5)	M (7.6)	SD (7.3)
Max (4.3)	Max (4.5)	Max (5.1)	M (5.0)	M (4.7)	M (4.7)	M (6.7)	Max (4.7)	Max (5.0)

*Table S4 - Number of samples used for modelling each active data outcome after standardization and removal of missing data points. PerfROs are marked with *.*

**Active data outcome**	**Representation type**	**Correlation coefficient**	**Final N of passive data points (% of the initially available)**
Anxiety	Levels	Pearson	216 (67.72 %)
		Spearman	215 (67.4 %)
		Kendall	215 (67.4 %)
	ΔABS	Pearson	131 (28.48 %)
		Spearman	131 (28.48 %)
		Kendall	131 (28.48 %)
	Δ%	Pearson	52 (11.31 %)
		Spearman	52 (11.31 %)
		Kendall	52 (11.31 %)
Cognitive decline	Levels	Pearson	215 (66.98 %)
		Spearman	215 (66.98 %)
		Kendall	215 (66.98 %)
	ΔABS	Pearson	134 (29.2 %)
		Spearman	134 (29.2 %)
		Kendall	134 (29.2 %)
	Δ%	Pearson	53 (11.55 %)
		Spearman	53 (11.55 %)
		Kendall	53 (11.55 %)
Depression	Levels	Pearson	213 (66.78 %)
		Spearman	213 (66.78 %)
		Kendall	213 (66.78 %)
	ΔABS	Pearson	129 (28.05 %)
		Spearman	129 (28.05 %)
		Kendall	129 (28.05 %)
	Δ%	Pearson	46 (10.65 %)
		Spearman	46 (10.65 %)
		Kendall	46 (10.65 %)

Hostility	Levels	Pearson	223 (67.58 %)
		Spearman	223 (67.58 %)
		Kendall	223 (67.58 %)
	ΔABS	Pearson	133 (28.79 %)
		Spearman	133 (28.79 %)
		Kendall	133 (28.79 %)
	Δ%	Pearson	22 (10.14 %)
		Spearman	22 (10.14 %)
		Kendall	22 (10.14 %)
Memory complaints	Levels	Pearson	213 (66.57 %)
		Spearman	214 (66.88 %)
		Kendall	213 (66.57 %)
	ΔABS	Pearson	135 (29.23 %)
		Spearman	135 (29.23 %)
		Kendall	135 (29.23 %)
	Δ%	Pearson	53 (11.48 %)
		Spearman	51 (11.04 %)
		Kendall	51 (11.04 %)
Negative affect	Levels	Pearson	213 (67.2 %)
		Spearman	213 (67.2 %)
		Kendall	213 (67.2 %)
	ΔABS	Pearson	133 (29.17 %)
		Spearman	133 (29.17 %)
		Kendall	133 (29.17 %)
	Δ%	Pearson	52 (11.41 %)
		Spearman	52 (11.41 %)
		Kendall	52 (11.41 %)
Positive affect	Levels	Pearson	213 (67.2 %)
		Spearman	213 (67.2 %)
		Kendall	213 (67.2 %)
	ΔABS	Pearson	134 (29.39 %)
		Spearman	132 (28.95 %)
		Kendall	132 (28.95 %)
	Δ%	Pearson	52 (11.41 %)
		Spearman	51 (11.19 %)
		Kendall	51 (11.19 %)
Stress	Levels	Pearson	212 (66.46 %)
		Spearman	210 (65.84 %)
		Kendall	210 (65.84 %)
	ΔABS	Pearson	132 (28.7 %)
		Spearman	132 (28.7 %)
		Kendall	132 (28.7 %)
	Δ%	Pearson	53 (11.53 %)
		Spearman	53 (11.53 %)
		Kendall	53 (11.53 %)
Attention	Levels	Pearson	221 (68.64 %)
		Spearman	214 (66.46 %)
		Kendall	214 (66.46 %)
	ΔABS	Pearson	134 (29.33 %)
		Spearman	132 (28.89 %)
		Kendall	132 (28.89 %)
	Δ%	Pearson	53 (11.63 %)
		Spearman	52 (11.41 %)

		Kendall	52 (11.41 %)
Cognitive flexibility *	Levels	Pearson	217 (67.61 %)
		Spearman	216 (67.29 %)
		Kendall	216 (67.29 %)
	ΔABS	Pearson	135 (29.87 %)
		Spearman	135 (29.87 %)
		Kendall	135 (29.87 %)
	Δ%	Pearson	55 (12.17 %)
		Spearman	55 (12.17 %)
		Kendall	55 (12.17 %)
Inductive reasoning *	Levels	Pearson	214 (69.04 %)
		Spearman	214 (69.04 %)
		Kendall	214 (69.04 %)
	ΔABS	Pearson	128 (29.16 %)
		Spearman	127 (28.93 %)
		Kendall	127 (28.93 %)
	Δ%	Pearson	49 (11.17 %)
		Spearman	49 (11.17 %)
		Kendall	49 (11.17 %)
Inhibitory control *	Levels	Pearson	216 (67.93 %)
		Spearman	216 (67.93 %)
		Kendall	217 (68.24 %)
	ΔABS	Pearson	133 (29.11 %)
		Spearman	134 (29.33 %)
		Kendall	134 (29.33 %)
	Δ%	Pearson	53 (11.6 %)
		Spearman	51 (11.16 %)
		Kendall	51 (11.16 %)
Long-term memory *	Levels	Pearson	211 (68.07 %)
		Spearman	212 (68.39 %)
		Kendall	212 (68.39 %)
	ΔABS	Pearson	126 (28.71 %)
		Spearman	126 (28.71 %)
		Kendall	126 (28.71 %)
	Δ%	Pearson	48 (11.19 %)
		Spearman	50 (11.66 %)
		Kendall	50 (11.66 %)
Processing speed *	Levels	Pearson	215 (66.98 %)
		Spearman	217 (67.61 %)
		Kendall	217 (67.61 %)
	ΔABS	Pearson	129 (28.54 %)
		Spearman	130 (28.77 %)
		Kendall	130 (28.77 %)
	Δ%	Pearson	52 (11.51 %)
		Spearman	51 (11.29 %)
		Kendall	51 (11.29 %)
Prospective memory *	Levels	Pearson	210 (67.75 %)
		Spearman	213 (68.71 %)
		Kendall	213 (68.71 %)
	ΔABS	Pearson	124 (28.25 %)
		Spearman	126 (28.71 %)
		Kendall	126 (28.71 %)
	Δ%	Pearson	34 (13.29 %)

		Spearman	37 (14.46 %)
		Kendall	37 (14.46 %)
Short-term memory *	Levels	Pearson	210 (67.75 %)
		Spearman	211 (68.07 %)
		Kendall	211 (68.07 %)
	ΔABS	Pearson	129 (29.39 %)
		Spearman	129 (29.39 %)
		Kendall	129 (29.39 %)
	Δ%	Pearson	49 (11.43 %)
		Spearman	49 (11.43 %)
		Kendall	49 (11.43 %)
Tapping speed *	Levels	Pearson	217 (68.89 %)
		Spearman	216 (68.58 %)
		Kendall	216 (68.58 %)
	ΔABS	Pearson	130 (28.51 %)
		Spearman	130 (28.51 %)
		Kendall	130 (28.51 %)
	Δ%	Pearson	51 (11.26 %)
		Spearman	52 (11.48 %)
		Kendall	52 (11.48 %)
Typing speed *	Levels	Pearson	212 (66.46 %)
		Spearman	213 (66.78 %)
		Kendall	214 (67.09 %)
	ΔABS	Pearson	129 (28.99 %)
		Spearman	129 (28.99 %)
		Kendall	129 (28.99 %)
	Δ%	Pearson	50 (11.24 %)
		Spearman	50 (11.24 %)
		Kendall	50 (11.24 %)
Verbal fluency 1 *	Levels	Pearson	209 (67.42 %)
		Spearman	209 (67.42 %)
		Kendall	209 (67.42 %)
	ΔABS	Pearson	127 (28.93 %)
		Spearman	127 (28.93 %)
		Kendall	127 (28.93 %)
	Δ%	Pearson	47 (10.81 %)
		Spearman	47 (10.81 %)
		Kendall	47 (10.81 %)
Verbal fluency 2 *	Levels	Pearson	208 (67.1 %)
		Spearman	211 (68.07 %)
		Kendall	211 (68.07 %)
	ΔABS	Pearson	129 (29.39 %)
		Spearman	128 (29.16 %)
		Kendall	128 (29.16 %)
	Δ%	Pearson	49 (12.22 %)
		Spearman	49 (12.22 %)
		Kendall	49 (12.22 %)
Working memory *	Levels	Pearson	213 (68.71 %)
		Spearman	212 (68.39 %)
		Kendall	212 (68.39 %)
	ΔABS	Pearson	132 (30.07 %)
		Spearman	129 (29.39 %)
		Kendall	129 (29.39 %)

	Δ%	Pearson	49 (11.32 %)
		Spearman	49 (11.32 %)
		Kendall	49 (11.32 %)

*Table S5 – Comparison of prediction errors between the population mean predictor (naïve model) and the machine learning model achieving the lowest SMAE. The table also includes the SMAE and SD differences between the two approaches (model − naïve). Lower difference values indicate better predictive performance relative to the naïve baseline. For all outcomes, we additionally report the p-value obtained from comparing paired fold-level errors between the two models, using a paired t-test or Wilcoxon signed-rank test depending on the normality of paired differences (Shapiro–Wilk test). PerfROs are marked with *.*

**Outcome**	**Repres.**	**Naïve model SMAE (SD)**	**Model with lowest M SMAE (SD) User CV**	**Comp. SMAE**	**Comp. SD**	**Test applied**	**p-value**
Attention *	Levels	17.27 % ± 32.18 %	15.32 % ± 21.67 %	- 1.95 %	- 10.51 %	Paired T-test	**3.29 x 10^{-6}**
Cognitive decline	ΔABS	3.27 % ± 2.83 %	3.22 % ± 2.15 %	- 0.05 %	- 0.68 %	Paired T-test	0.815
Cognitive flexibility *	Levels	16.22 % ± 22.48 %	14.98 % ± 15.59 %	- 1.24 %	- 6.89 %	Wilcoxon sig. rank	**0.036**
Inductive reasoning *	Levels	14.79 % ± 10.86 %	13.45 % ± 7.28 %	- 1.34 %	- 3.58 %	Wilcoxon sig. rank	0.063
Inhibitory control *	Levels	9.25 % ± 30.39 %	7.51 % ± 8.36 %	- 1.74 %	- 22.03 %	Wilcoxon sig. rank	0.453
Long-term memory *	Levels	18.50 % ± 12.87 %	16.39 % ± 7.24 %	- 2.11 %	- 5.63 %	Wilcoxon sig. rank	0.109
Memory	ΔABS	8.47 % ± 6.72 %	6.45 % ± 5.11 %	- 2.02 %	- 1.61 %	Paired T-test	0.293
Processing speed *	Levels	20.11 % ± 27.69 %	19.06 % ± 21.26 %	- 1.05 %	- 6.43 %	Wilcoxon sig. rank	0.207
Prospective memory *	ΔABS	14.69 % ± 19.59 %	15.14 % ± 15.64 %	+ 0.45 %	- 3.95 %	Wilcoxon sig. rank	0.997
Short-term memory *	Levels	17.02 % ± 11.96 %	16.41 % ± 7.40 %	- 0.61 %	- 4.56 %	Paired T-test	0.284
Tapping speed *	ΔABS	10.42 % ± 11.24 %	10.13 % ± 9.39 %	- 0.29 %	- 1.85 %	Paired T-test	0.838
Typing speed *	ΔABS	10.92 % ± 11.07 %	9.57 % ± 7.31 %	- 1.35 %	- 3.76 %	Paired T-test	0.940
Verbal fluency 1 *	ΔABS	17.71 % ± 18.88 %	14.03 % ± 10.72 %	- 3.68 %	- 8.16 %	Paired T-test	**0.045**
Verbal fluency 2 *	Δ%	38.87 % ± 88.06 %	25.33 % ± 18.23 %	- 13.54 %	- 69.83 %	Wilcoxon sig. rank	0.808
Working memory *	ΔABS	16.58 % ± 16.94 %	15.36 % ± 13.93 %	- 1.22 %	- 3.01 %	Wilcoxon sig. rank	0.136
Anxiety	ΔABS	7.79 % ± 5.95 %	7.30 % ± 4.49 %	- 0.49 %	- 1.46 %	Paired T-test	0.883
Depression	ΔABS	8.59 % ± 7.70 %	7.78 % ± 4.57 %	- 0.81 %	- 3.13 %	Paired T-test	0.235
Hostility	ΔABS	8.55 % ± 12.47 %	12.68 % ± 11.48 %	+ 4.13 %	- 0.99 %	Wilcoxon sig. rank	**0.016**
Negative affect	ΔABS	10.18 % ± 9.74 %	9.88 % ± 8.36 %	- 0.30 %	- 1.38 %	Paired T-test	0.300
Positive affect	Δ%	16.16 % ± 15.68 %	12.14 % ± 4.59 %	- 4.02 %	- 11.09 %	Paired T-test	0.844

Stress	ΔABS	8.90 % ± 7.50 %	8.10 % ± 5.70 %	- 0.80 %	- 1.80 %	Paired T-test	0.245

*Table S6 – Mean and SD overlap coefficients quantifying the proportion of features selected in the first modelling round that would have remained selected if each participant was excluded per fold (leave-one-out analysis). Values close to 1.0 indicate stable feature selection across individuals. PerfROs are marked with *.*

Outcome	M SMAE user	Mdn SMAE user
Attention *	0.987 ± 0.032	0.997 ± 0.028
Cognitive decline	0.987 ± 0.033	1.000 ± 0.000
Cognitive flexibility *	0.995 ± 0.012	0.995 ± 0.012
Inductive reasoning *	0.998 ± 0.008	0.998 ± 0.008
Inhibitory control *	0.998 ± 0.014	0.998 ± 0.014
Long-term memory *	0.992 ± 0.015	0.992 ± 0.015
Memory	1.000 ± 0.000	1.000 ± 0.000
Processing speed *	0.986 ± 0.017	0.986 ± 0.017
Prospective memory *	0.995 ± 0.016	0.995 ± 0.016
Short-term memory *	0.986 ± 0.020	0.992 ± 0.013
Tapping speed *	0.991 ± 0.022	0.975 ± 0.033
Typing speed *	0.994 ± 0.011	0.994 ± 0.011
Verbal fluency 1 *	1.000 ± 0.000	0.958 ± 0.045
Verbal fluency 2 *	1.000 ± 0.000	0.998 ± 0.007
Working memory *	0.974 ± 0.019	0.984 ± 0.018
Anxiety	0.999 ± 0.004	0.999 ± 0.004
Depression	0.998 ± 0.011	0.998 ± 0.011
Hostility	0.994 ± 0.013	1.000 ± 0.000
Negative affect	0.998 ± 0.008	0.998 ± 0.008
Positive affect	0.973 ± 0.029	1.000 ± 0.000
Stress	0.995 ± 0.011	0.995 ± 0.011

*Table S7 – Mean and SD Jaccard indices comparing the set of features selected in the first modelling round that would have remained selected if each participant was excluded per fold (leave-one-out analysis). The Jaccard index accounts for both shared and unique features, providing a complementary and conservative measure of feature selection robustness. PerfROs are marked with *.*

Outcome	M SMAE user	Mdn SMAE user
Attention *	0.976 ± 0.067	0.993 ± 0.062
Cognitive decline	0.976 ± 0.052	1.000 ± 0.000
Cognitive flexibility *	0.994 ± 0.015	0.994 ± 0.015
Inductive reasoning *	0.984 ± 0.020	0.984 ± 0.020
Inhibitory control *	0.995 ± 0.034	0.995 ± 0.034
Long-term memory *	0.986 ± 0.026	0.986 ± 0.026
Memory	1.000 ± 0.000	1.000 ± 0.000
Processing speed *	0.974 ± 0.030	0.974 ± 0.030
Prospective memory *	0.988 ± 0.030	0.988 ± 0.030
Short-term memory *	0.972 ± 0.038	0.984 ± 0.025
Tapping speed *	0.987 ± 0.040	0.958 ± 0.072
Typing speed *	0.988 ± 0.022	0.988 ± 0.022
Verbal fluency 1 *	1.000 ± 0.000	0.943 ± 0.064
Verbal fluency 2 *	0.973 ± 0.031	0.998 ± 0.007
Working memory *	0.965 ± 0.029	0.966 ± 0.035
Anxiety	0.999 ± 0.004	0.999 ± 0.004

Depression	0.996 ± 0.016	0.996 ± 0.016
Hostility	0.988 ± 0.027	1.000 ± 0.000
Negative affect	0.995 ± 0.017	0.995 ± 0.017
Positive affect	0.951 ± 0.053	1.000 ± 0.000
Stress	0.990 ± 0.021	0.990 ± 0.021

*Table S8 – Top three predictors contributing most to the prediction of each cognitive and affective outcome, based on mean feature importance values from the model with the lowest SMAE under user-dependent cross-validation. Vertical separators distinguish cognitive outcomes from affective outcomes. Rows list all predictors, grouped by domain (e.g., physical activity, sleep, and control variables). Each cell indicates which statistical representation (e.g., Mean, SD, IQR, Skew, Kurtosis) of the corresponding predictor ranked among the top three for that outcome, as shown in the legend below the table. PerfROs are marked with *. Units of the predictors are specified in Table S9.*

**Predictors** \ **Outcome**	**Attention ***	**Cognitive decline**	**Cognitive flexibility ***	**Inductive reasoning ***	**Inhibitory control ***	**Long-term memory ***	**Memory**	**Processing speed ***	**Prospective memory**	**Short-term memory ***	**Tapping speed ***	**Typing speed ***	**Verbal fluency 1 ***	**Verbal fluency 2 ***	**Working memory ***	**Anxiety**	**Depression**	**Hostility**	**Negative affect**	**Positive affect**	**Stress**
Active calories b.							$												$	$	
Deep sleep dur.					K					-											
Sleep efficiency											$	$									
Sleep score															x̄						
Wake-up count				S																	
Wake-up latency	K		$																		
Sleeping HR M															x̃						
Sleeping HR Min								-													
24-hour HR M														R				S			
24-hour HR SD	S					K	S					K									$
24-hour HR Min		x̃												R							R
24-hour HR Max												S									
Air temperature		K									+										
Atmosp. pressure													R	+							
Humidity				-				K								-			-		
Max air temp. f.											+		-								
Min air temp for.					x̄				+	K								S		+	
Temp. feeling			K						+									S			
Ammonia				-			R														
Carbon monoxide						-				-						-					
Ozone		$															R				
Nitrogen dioxide			$					$							+						
PM 10																	-				
Sulfur dioxide					$											K					x̃
Wear time perc.													S								
Subj. age diff.	X																		X		
Time difference									X								X				
Past smoker						X															
White wine cons.																				X	

x̄	x̃	$	R	-	+	S	K	X
Mean	Median	SD	IQR	Min	Max	Skew	Kurtosis	NA

Table S9 – Units of the passive data metrics collected.

**Passive data metric**	**Units**
Active calories burned	Kcal
Distance walking or running	Meters
Distance rate	Meters per wear second
Steps<br>Sleep score<br>Wake-up count	Not applicable
Step frequency	Steps per wear second
Deep sleep duration<br>Light sleep duration<br>Sleep latency<br>Time spent in bed<br>Total sleep time<br>Wake-up latency<br>Wakefulness after sleep onset	Seconds
Sleep efficiency<br>Humidity<br>Wear time day percentage	Percentage
Sleeping HR<br>24-hour HR	Beats per minute
Air temperature<br>Maximum air temperature forecasted<br>Minimum air temperature forecasted<br>Temperature feeling	Celsius
Atmospheric pressure	hPa
Air quality index	According to the *OpenWeather* index
Ammonia<br>Carbon monoxide<br>Ozone<br>Nitric oxide<br>Nitrogen dioxide<br>Particulate matter 2.5<br>Particulate matter 10<br>Sulfur dioxide	μg / meter ^{3}<br>(microgram per cubic meter)
Time zone difference	Hours

Supplementary Material B: Supplementary Figures

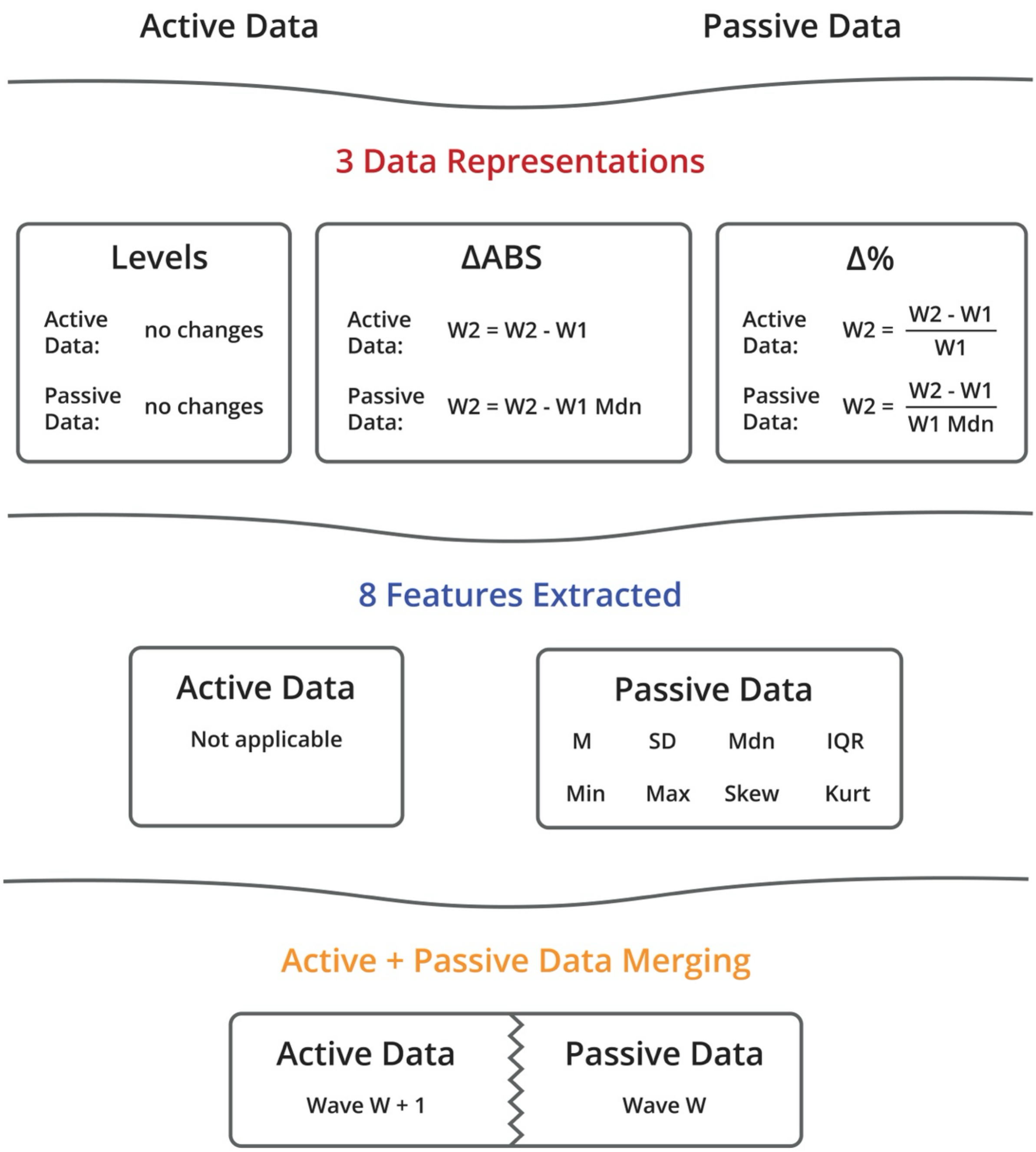

Figure S1 - Illustration of the different data representations, features extracted, and final merging of active with passive data.

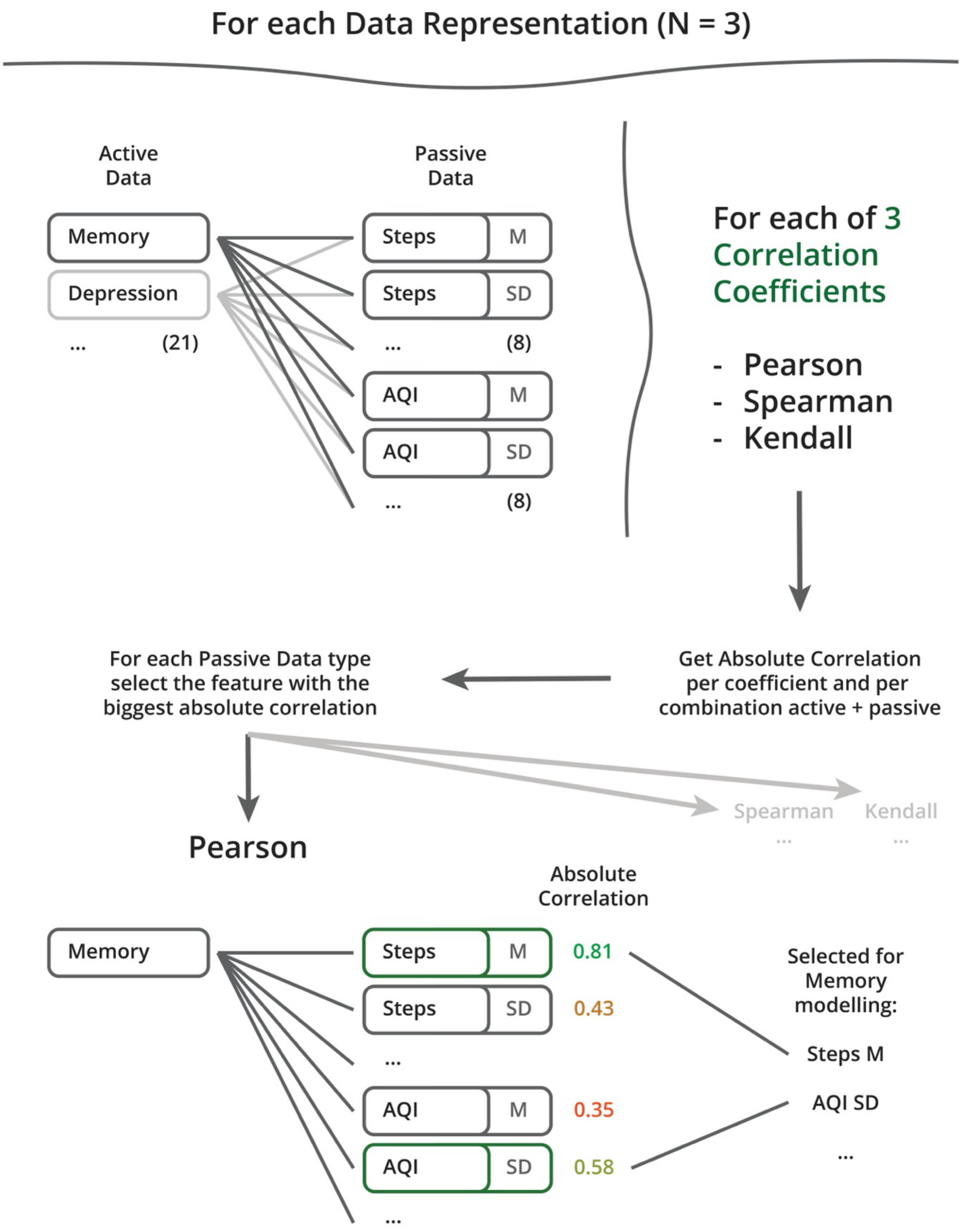

Figure S2 - Illustration of the feature selection process applied to each data representation and each correlation coefficient.

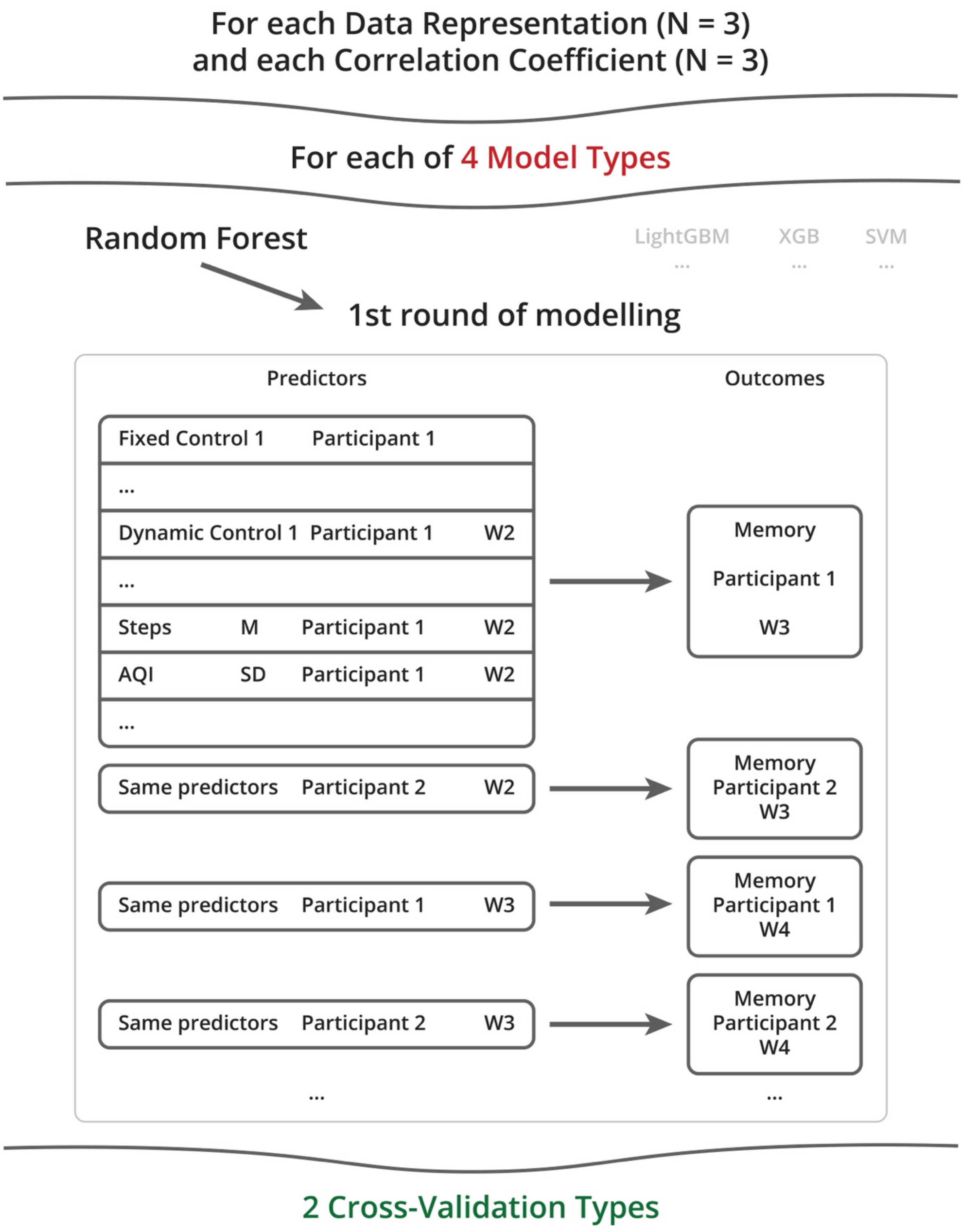

Figure S3 - Illustration of the steps undertaken for the first stage of modelling.

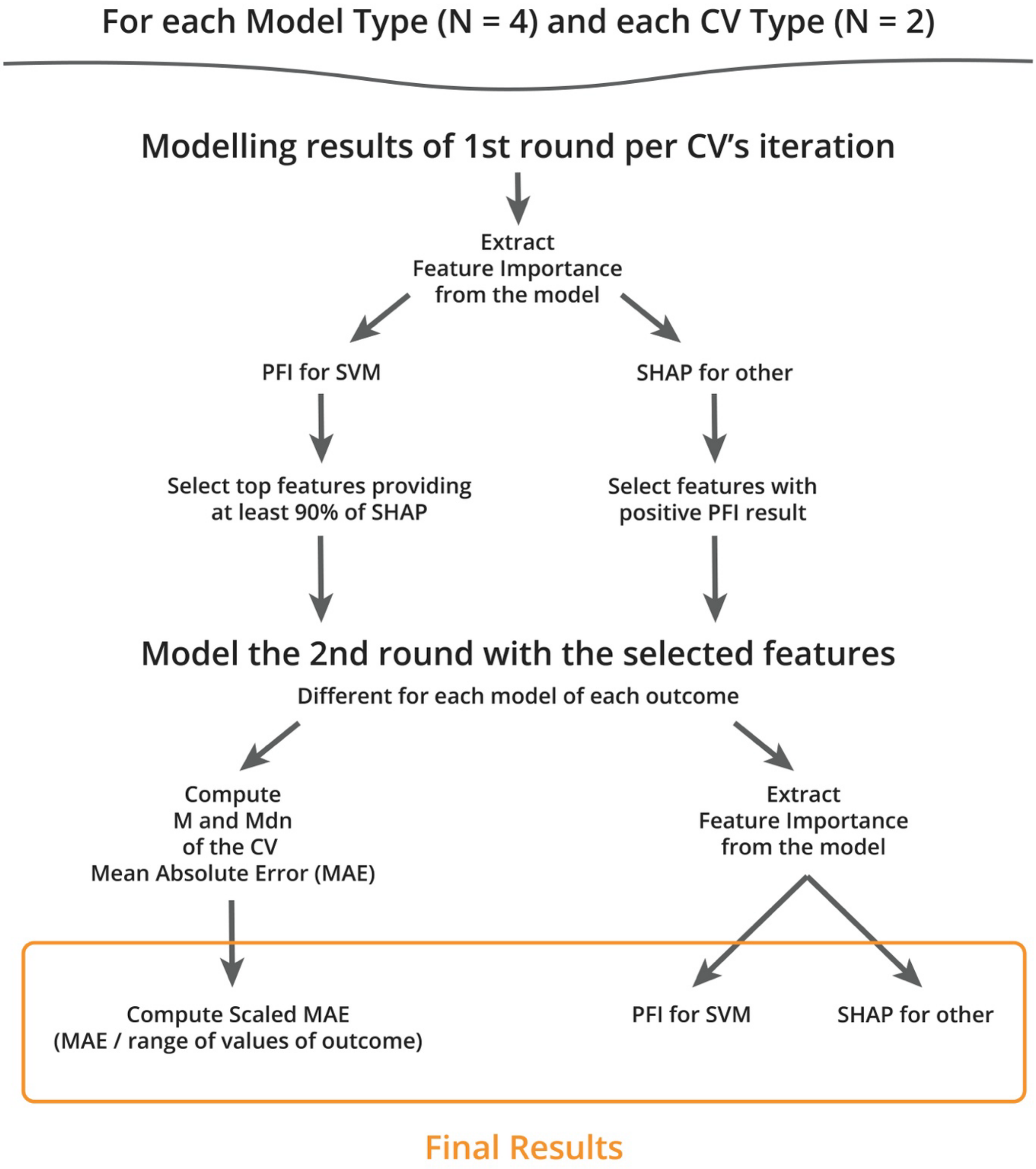

Figure S4 - Illustration of the steps between the first round of modelling and the final results of our research.

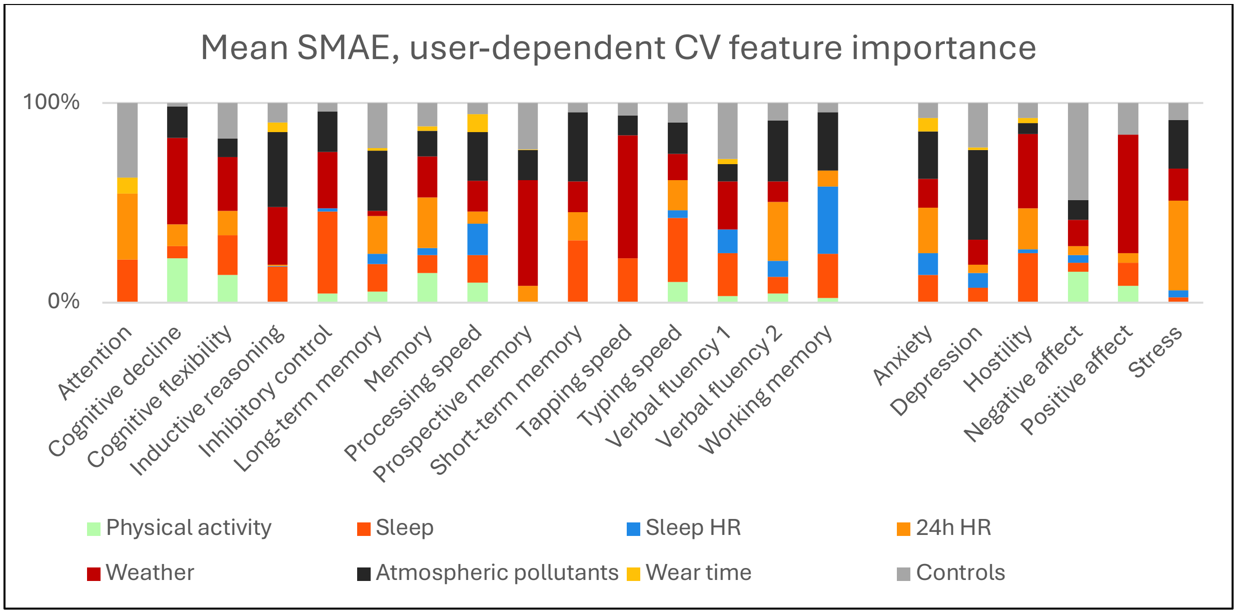

Figure S5 - Feature importance across groups of passive metrics for the most accurate models according to the Mean SMAE using user-dependent CV. The Cognition outcomes are on the left side of the chart, and the Affective States outcomes on the right.

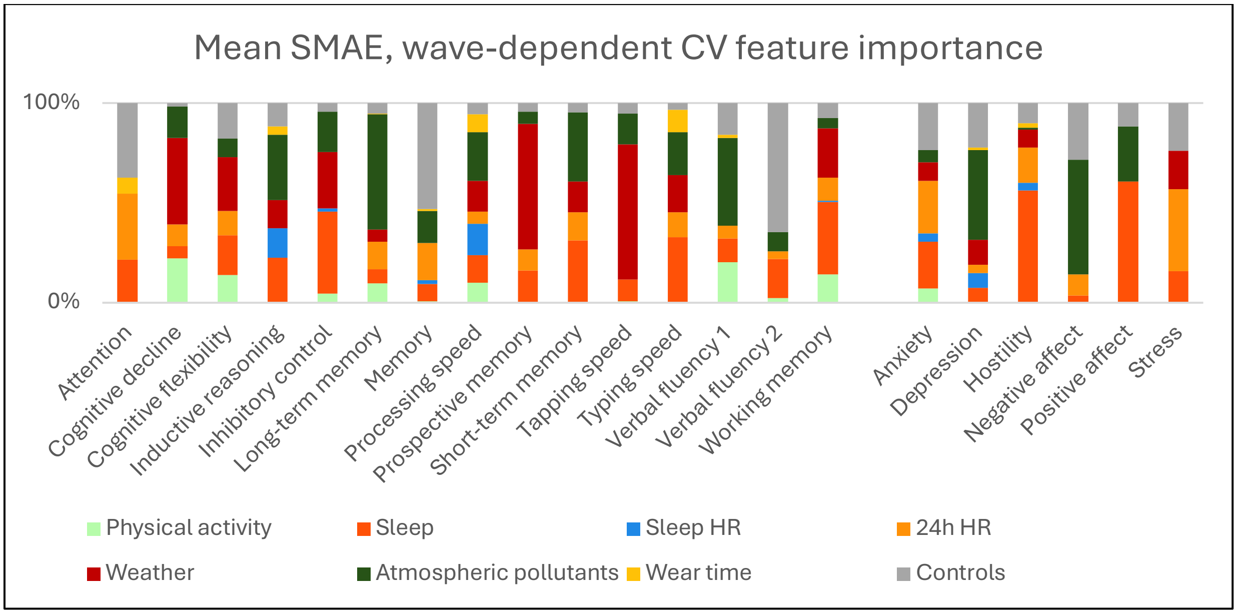

Figure S6 - Feature importance across groups of passive metrics for the most accurate models according to the Mean SMAE using wave-dependent CV. The Cognition outcomes are on the left side of the chart, and the Affective States outcomes on the right.

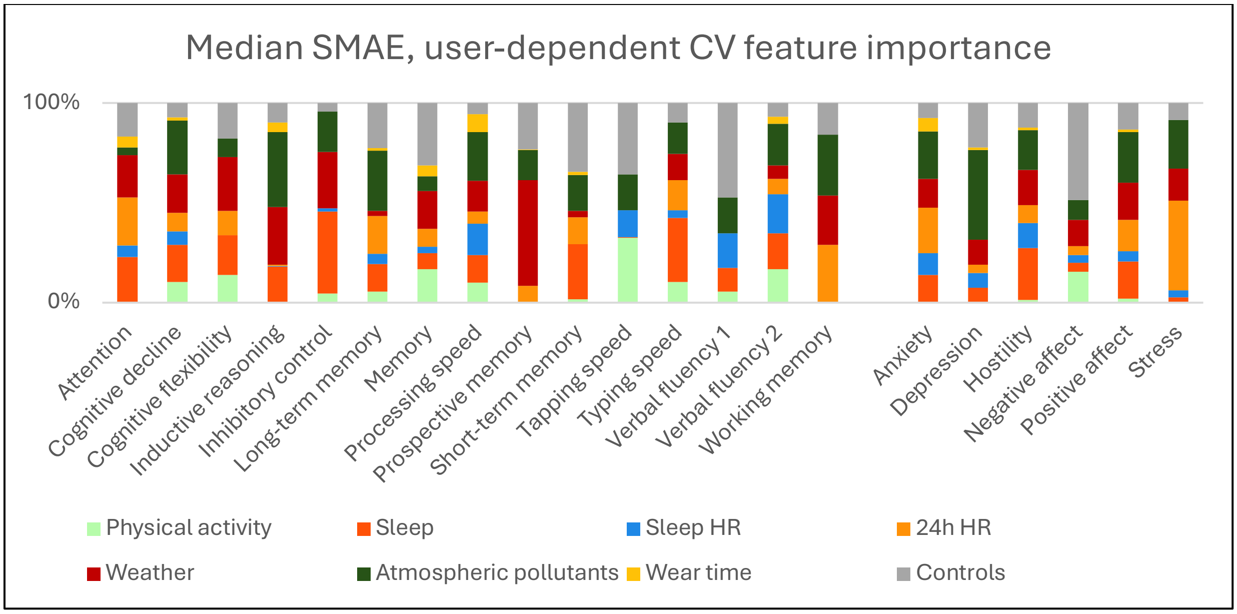

Figure S7 - Feature importance across groups of passive metrics for the most accurate models according to the Median SMAE using user-dependent CV. The Cognition outcomes are on the left side of the chart, and the Affective States outcomes on the right.

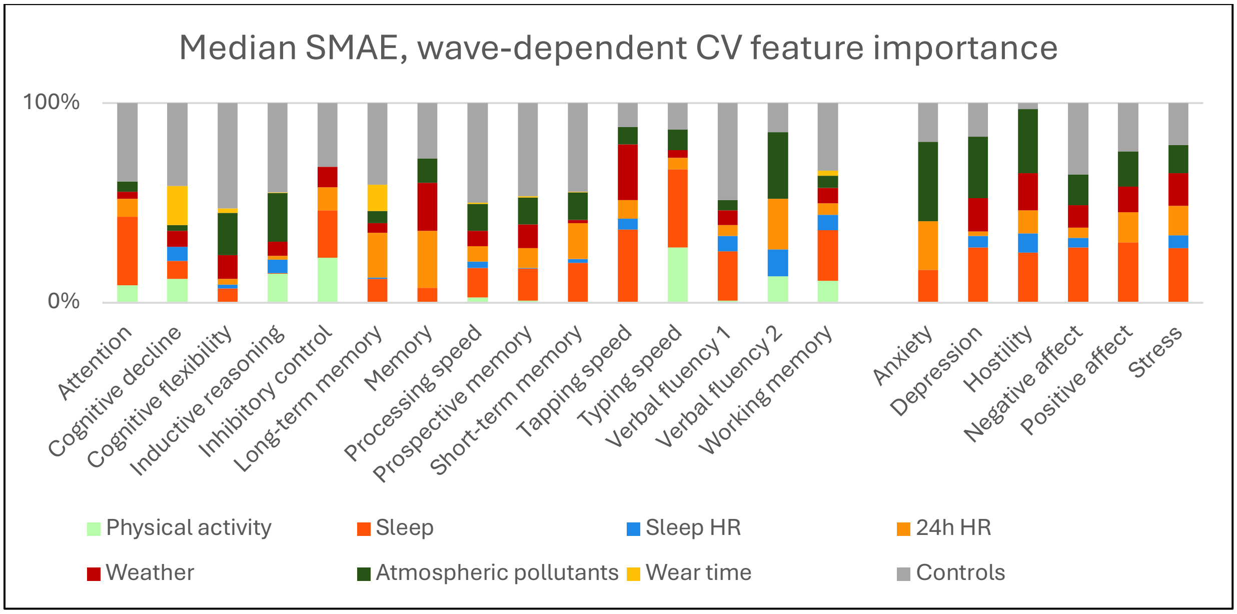

Figure S8 - Feature importance across groups of passive metrics for the most accurate models according to the Median SMAE using wave-dependent CV. The Cognition outcomes are on the left side of the chart, and the Affective States outcomes on the right.

Supplementary Material C: Other Supplementary Materials

Supplementary Material C 1: Description of data used after data exclusion.

Data-derived Metrics

The average combined task duration decreased over the study period, from around 47 minutes (wave 1, 16 tasks) to about 33 minutes (wave 2, 13 tasks) then to 30 minutes (wave 3, 13 tasks) and finally to around only 19 minutes (wave 4, 13 tasks). A *linear regression* (LR) fit explained 95% of its variance with a p-value of 0.025, achieving similar results even when adjusting for the number of tasks given in each wave. That can be explained in at least two different ways: (1) learning effects may have made the still enrolled participants become familiarized with the tasks, thus completing them in less time and/or reflecting for less time when answering PROs, and (2) as the number of total participants engaged decreases slowly wave after wave, the average level of engagement may have been increasing assuming that after some waves the individuals still participating are those with a motivation above average.

Passive Data Smartwatch-based

When looking at the valid days included in each wave, the average daily wear time per included participant is consistently above 23 hours and 10 minutes (as in Table S9), meaning that the data used in this research is either treated as missing or represents more than 96% of the day. An illustration of the spreading of valid days across waves and participants is provided in Figure S9.

Table S10 - Valid days and valid hours per participant included in each wave's analysis.

Wave	Maximum days (*max*)	Participants included (/ 82)	Valid days per participant		Wear time per valid day per participant (hh:mm:ss)	
			M / *max*	SD / *max*	M (/ 24 h)	SD (/ 24 h)
1	97	57 (0.70)	0.85	0.16	23:18:54 (0.97)	01:27:00 (0.06)
2	93	75 (0.92)	0.96	0.08	23:10:50 (0.97)	01:29:43 (0.06)
3	90	77 (0.94)	0.96	0.10	23:12:31 (0.97)	01:28:58 (0.06)
4	33	73 (0.89)	0.94	0.12	22:10:51 (0.92)	01:46:56 (0.07)

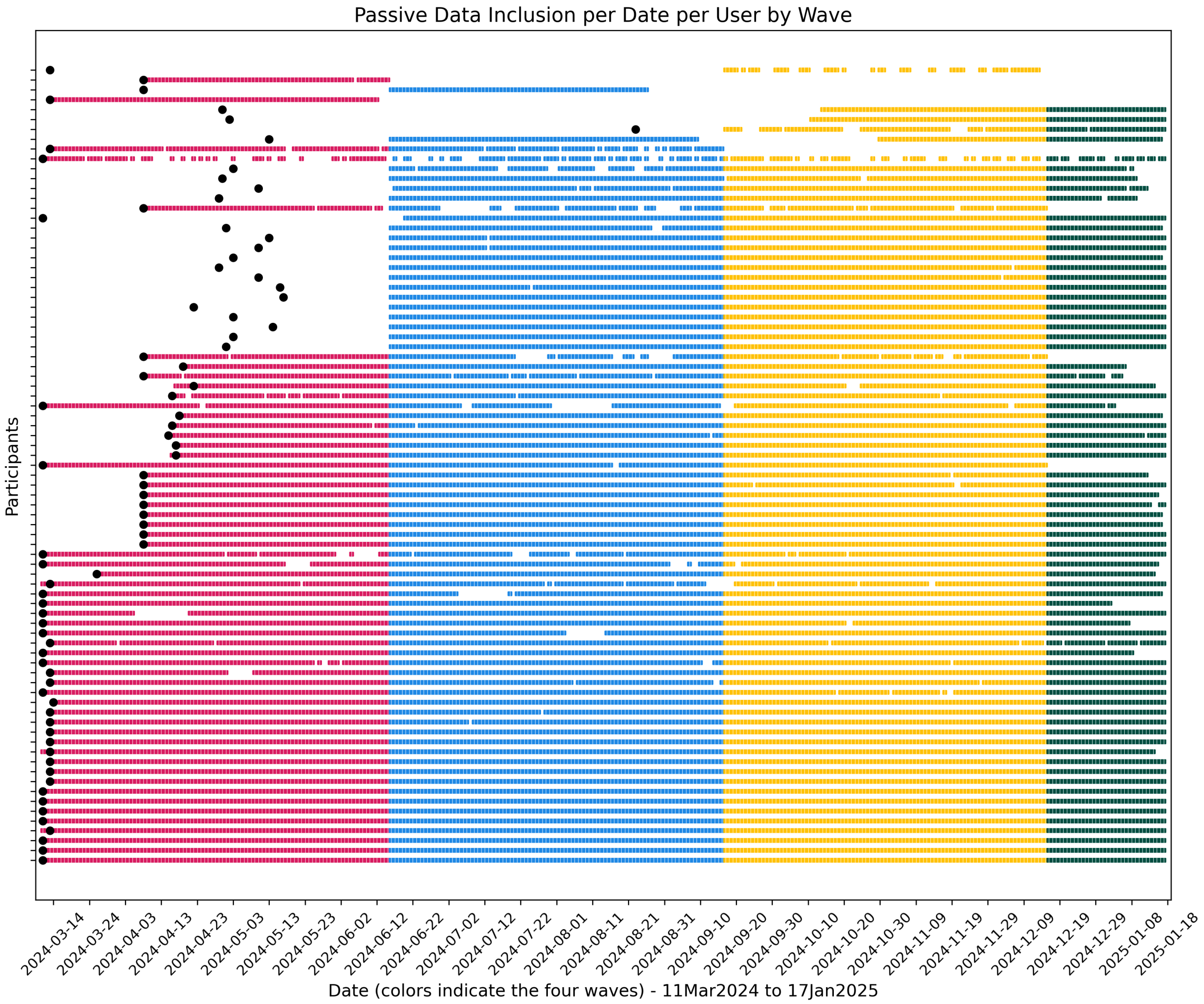

Figure S9 - Days included in the analysis across waves and per participant. The black dots represent the enrolment date of each participant being plotted.

Passive Data mQoL app-based

As detailed in the Method section, a minimum data criterion was applied considering at the data on weather and atmospheric pollutants. The scope of this final data exclusion round was to limit the pool of participants included to those with minimum atmospheric data collected, regardless of whether they had been selected based on the smartwatch-based approach. As presented by Table S10, the number of participants with at least one record of these data points remained around 75 across the four waves. However, the average number of measurements per participant decreased between waves 1 and 3, increasing again for wave 4. That decrease and subsequent increase can be observed in all metrics. Despite that, the lowest average sampling frequency was every 9 days in wave 3, with the lowest being only once in all waves 3 and 4. Despite once per wave not being the optimal sampling of such environment information, it allows us to at least estimate the effect of weather and air pollutants on the moment of assessment of the cognition and affective state outcomes.

Table S11 - Number of atmospheric data points collected per participant per wave.

**Wave**	**Max days (*max*)**	**Data type**	**Participants included (/ 82)**	**Meas. per participant per day (≈ every days)**				
				**M / *max***	**SD / *max***	**Mdn / *max***	**Min / *max***	**Max / *max***
1	97	Weather	78 (0.95)	0.28 (4)	0.16 (7)	0.25 (4)	0.02 (49)	1.20 (1)
		Air pollutants		0.26 (4)	0.15 (6)	0.24 (4)	0.02 (49)	1.15 (1)
2	93	Weather	76 (0.93)	0.19 (5)	0.13 (8)	0.15 (7)	0.04 (23)	0.56 (2)
		Air pollutants		0.17 (6)	0.12 (9)	0.14 (7)	0.04 (23)	0.54 (2)
3	90	Weather	78 (0.95)	0.11 (9)	0.08 (13)	0.09 (11)	0.01 (90)	0.47 (2)
		Air pollutants		0.11 (9)	0.07 (13)	0.09 (11)	0.01 (90)	0.44 (2)
4	33	Weather	74 (0.90)	0.30 (4)	0.24 (5)	0.24 (4)	0.03 (33)	1.76 (1)
		Air pollutants		0.28 (3)	0.22 (4)	0.24 (4)	0.03 (33)	1.64 (1)

Supplementary Material C 2: Descriptive analysis of the metrics computed.

Controls

Regarding the control variables, Table S11 includes the details for the variables only collected at the baseline.

Table S12 - Metrics and trend analysis of control variables measured only at baseline.

Control variable	M ± SD	Mdn
BMI at the age of 40	23.58 ± 4.66	22.34
Total CRIQ score	127.93 ± 18.62	125.50
Education CRIQ score	124.01 ± 23.44	213.00
Working activity CRIQ score	119.63 ± 17.21	117.50
Leisure time CRIQ score	119.77 ± 22.99	117.50
MDS total score	32.77 ± 4.06	33.00

As for the recurrently collected controls, overall, none of them showed a significant trend (by fitting a Linear Regression to the data) over the four waves used in this research, as presented by Table S12.

Table S13 - Metrics and trend analysis of control variables measured across waves, with beta and p-value of a linear regression (LR) fit.

Control variable	M ± SD	Mdn	LR beta	LR p-value
Chronological age at each wave's moment	58.00 ± 0.49	57.75	+ 0.36	0.402
Subjective age difference	7.87 ± 0.46	6.75	+ 0.31	0.431
Occurrence of head injuries in the past	0.18 ± 0.04	0.00	- 0.03	0.121
Education years (each wave value)	17.72 ± 0.53	17.50	- 0.07	0.800
Frequent smoker in the past	0.40 ± 0.02	0.00	- 0.01	0.639
Frequent smoker in past wave	0.13 ± 0.03	0.00	- 0.02	0.236
Diagnosis cardiovascular dis.	0.06 ± 0.01	0.00	0.00	0.762
Diagnosis sleep disorders	0.15 ± 0.02	0.00	+ 0.01	0.710
Avg. red wine glasses per week	1.78 ± 0.07	1.00	+ 0.01	0.960
Avg. white wine glasses per week	1.19 ± 0.11	0.00	+ 0.08	0.433
Avg. glasses other alcohol per week	1.19 ± 0.11	1.00	- 0.01	0.919

Active Data

Like the metrics computed and modelled from the wave-dependent control variables, the active data points measuring PROs and PerfROs were analysed. Table S13 contains their results in the Levels representation, which indicate that four active data outcomes show a statistically significant trend over waves: long-term, prospective, and short-term memories, and verbal fluency version 1's performance improves over time.

*Table S14 - Metrics and trend analysis of active data absolute values measured across waves. NA stands for Not Applicable. PerfROs are marked with *.*

Active data outcome (Levels)	M ± SD	Mdn	Mode	LR beta	LR p-value
Anxiety	7.85 ± 0.34	8.00	6.00	- 0.15	0.248
Cognitive decline	3.10 ± 0.03	3.08	3.00	+ 0.01	0.580
Depression	4.00 ± 0.27	3.50	2.00	- 0.03	0.812
Hostility	0.70 ± 0.03	0.00	0.00	+ 0.01	0.880
Memory complaints	36.30 ± 0.68	35.00	29.00	- 0.02	0.971
Negative affect	17.36 ± 0.60	16.50	10.00	- 0.09	0.770
Positive affect	32.69 ± 0.64	33.00	33.00	+ 0.20	0.597
Stress	1.85 ± 0.06	1.85	2.00	+ 0.01	0.671
Attention *	837.74 ± 76.96	487.00	NA	- 11.76	0.842
Cognitive flexibility *	8.97 ± 1.03	6.00	5.00	- 0.58	0.206
Inductive reasoning *	5.59 ± 0.20	6.00	6.00	+ 0.15	0.075
Inhibitory control *	112.99 ± 61.36	54.56	NA	+ 29.01	0.357
Long-term memory *	5.04 ± 0.76	5.00	5.00	+ 0.52	**0.000**
Processing speed *	6.02 ± 0.19	5.00	4.00	+ 0.01	0.946
Prospective memory *	0.63 ± 0.13	1.00	1.00	+ 0.08	**0.001**
Short-term memory *	5.19 ± 0.85	5.00	7.00	+ 0.58	**0.000**
Tapping speed *	244.26 ± 10.90	257.00	NA	- 8.08	0.077
Typing speed *	1.72 ± 0.04	1.69	NA	+ 0.03	0.316
Verbal fluency 1 *	10.53 ± 1.69	11.00	NA	+ 0.99	**0.000**
Verbal fluency 2 *	9.02 ± 0.46	9.00	NA	+ 0.15	0.424
Working memory *	7.81 ± 0.56	8.50	11.00	+ 0.10	0.508

Regarding the ΔABS and Δ% representations of active data, Table S14 presents the results with a p-value less or equal to 0.05. Such data can be interpreted as the rate of change in each active data outcome over time. The ones found to be discernible and indicated in the table refer to anxiety (for which no trend was found in the absolute values) and to the units found to reveal a trend in the absolute values except the short-term memory. Looking at the anxiety data change's trend, this means that, although there was no constant increase or decrease of anxiety values across the study population, the rate of change of values over time was stable and increasing. As for long-term memory, the rate of change of values tended to also increase over time, meaning that on top of tending to increase performance over time, that increase rate also increased over waves, possibly linking to practice effects. Finally, prospective memory and verbal fluency revealed a decreasing speed of change in their values, meaning that although participant's performance in those two tasks increased over time, the speed with which they evolved slowed down over waves, potentially showing a reduced effect of practice at the end of the fourth wave compared to the second.

*Table S15 - Metrics and trend analysis of active data difference of values measured across waves and with a p-value of at most 0.05. PerfROs are marked with *.*

Active data outcome (representation type)	M ± SD	Mdn	LR beta	LR p-value
Anxiety (ΔABS)	- 0.10 ± 0.49	0.00	+ 0.42	**0.015**
Anxiety (Δ%)	2.62 ± 6.95	0.00	+ 5.44	**0.026**
Long-term memory * (ΔABS)	0.64 ± 0.75	1.00	+ 0.37	**0.045**
Prospective memory * (ΔABS)	0.07 ± 0.15	0.00	- 0.11	**0.007**

Verbal fluency 1 * (ΔABS)	0.72 ± 2.01	0.00	- 1.28	**0.001**
Verbal fluency 1 * (Δ%)	72.28 ± 97.61	0.00	- 94.96	**0.000**

Passive Data

Eight features were extracted to describe the evolution of each of the 40 passive data units on each wave. To better understand the results of modelling ahead in this article, we first need to analyse the evolution of each of those metrics along the four waves of data. Table S15 presents the M, SD, and Mdn of each combination of passive data type and feature for the Levels data representation across the four waves and the result of using an LR model to fit its data.

We can observe some trends. Regarding physiology, first, motion-related data (calories, distance, steps) showed a significant negative trend over time, meaning a decrease in the average physical practice over waves. Such a trend was also observed in the frequency of extreme values over time (note the Kurt value's evolution for distance and steps, which decreased over time indicating less outliers by the end of the data collection period). Second, 24-hour HR tended to decrease over time overall (M and Mdn), in variation (SD and IQR), and in frequency of extreme values distribution (Kurt). However, the 24h minimum HR values tended to increase slightly. Third, sleep time increased slightly over time, which can be linked evenly to a similarly observed increase in light and deep sleep durations. Aligned with that, the sleep score tended to increase, but contrarily, the WASO increased in variability. Additionally, wake-up latency tended to decrease. Lastly, sleeping HR only revealed a trend regarding its maximum values over time, slightly decreasing their variability.

Table S16 - Metrics and trend analysis of passive data Levels measured across waves. P-values at most 0.05, and the lowest p-value result is in the case of no significant ones for the data type. Units are specified in Table S9.

	**Passive data type (Levels)**	**Metric**	**M ± SD**	**Mdn**	**LR beta**	**LR p-value**
Physical activity	Active calories	Max	387.56 ± 249.62	324.48	- 27.50	**0.020**
	Distance	Max	12421.76 ± 6517.14	10962.45	- 901.90	**0.002**
		Kurt	1.20 ± 2.43	0.47	- 0.25	**0.027**
	Distance rate	Max	0.15 ± 0.08	0.14	- 0.01	**0.002**
	Steps	Max	16566.49 ± 8167.95	14145.00	- 1185.70	**0.001**
		Kurt	1.08 ± 2.03	0.39	- 0.24	**0.030**
	Step frequency	Max	0.21 ± 0.10	0.18	- 0.01	**0.003**
		Kurt	1.21 ± 1.96	0.67	- 0.25	**0.021**
Sleep	Deep sleep duration	Min	5715.96 ± 2519.50	5460.00	+ 267.59	**0.049**
	Light sleep duration	Min	6324.30 ± 2526.16	6540.00	+ 307.47	**0.029**
	Sleep efficiency	Min	0.84 ± 0.11	0.86	+ 0.01	0.051
	Sleep latency	Skew	4.69 ± 1.85	4.23	- 0.44	**0.000**
		Kurt	26.26 ± 20.76	18.02	- 4.44	**0.000**
	Sleep score (proprietary algorithm)	Min	25.99 ± 13.70	20.00	+ 3.38	**0.000**
		Skew	- 1.63 ± 0.93	- 1.62	+ 0.11	**0.027**
		Kurt	3.68 ± 5.15	2.23	- 0.49	**0.044**
	Time spent in bed	Min	15277.85 ± 4090.51	14760.00	+ 882.81	**0.000**
		Kurt	0.95 ± 2.18	0.46	- 0.27	**0.023**
		Skew	- 0.17 ± 0.71	- 0.15	+ 0.08	**0.024**
		M	28394.25 ± 3269.13	28130.32	+ 396.57	**0.042**
	Total sleep time	Min	14440.25 ± 3889.00	14040.00	+ 785.65	**0.000**
		Kurt	0.90 ± 2.13	0.41	- 0.27	**0.019**
		M	26882.58 ± 3108.05	26743.64	+ 327.04	**0.042**
		Skew	- 0.24 ± 0.69	- 0.24	+ 0.07	**0.043**
	Wake-up count	SD	1.33 ± 0.51	1.23	+ 0.05	0.126

	Wake-up latency	Max	1354.18 ± 879.65	1140.00	- 169.62	**0.000**
		SD	278.29 ± 189.01	240.22	- 23.84	**0.003**
		Skew	2.66 ± 1.35	2.38	- 0.14	**0.028**
		Kurt	9.29 ± 11.52	5.66	- 1.16	**0.028**
		M	159.34 ± 122.70	118.55	- 10.96	**0.034**
	WASO	SD	1029.23 ± 601.08	874.17	+ 64.57	0.086
Sleep HR	Sleeping HR M	Min	56.84 ± 6.76	57.00	+ 0.62	0.078
	Sleeping HR Min	Skew	- 0.09 ± 0.95	0.03	- 0.07	0.187
	Sleeping HR Max	Max	126.11 ± 22.00	123.00	- 3.62	**0.003**
		Min	66.29 ± 8.39	67.00	+ 1.12	**0.012**
		Kurt	3.58 ± 8.39	1.45	- 0.81	**0.012**
		Skew	1.14 ± 1.25	0.87	- 0.12	**0.029**
24h HR	24h HR mean	Max	96.62 ± 14.47	92.39	- 2.52	**0.000**
		IQR	6.99 ± 5.85	5.63	- 0.70	**0.003**
		SD	6.16 ± 3.80	4.80	- 0.42	**0.015**
	24h HR standard deviation	Max	26.15 ± 8.39	23.05	- 1.11	**0.008**
		IQR	4.27 ± 2.48	3.61	- 0.22	**0.028**
		M	14.53 ± 2.88	14.21	- 0.26	**0.030**
		Mdn	13.93 ± 2.71	13.58	- 0.23	**0.036**
	24h HR median	Max	107.64 ± 28.58	98.50	- 4.29	**0.001**
		IQR	9.01 ± 11.46	5.75	- 1.26	**0.008**
		M	74.58 ± 9.85	73.63	- 0.97	**0.030**
		SD	9.50 ± 7.89	5.60	- 0.77	**0.036**
	24h HR minimum	Kurt	0.64 ± 2.39	- 0.03	- 0.33	**0.000**
		Min	35.58 ± 2.43	35.00	+ 0.33	**0.035**
	24h HR maximum	Max	201.88 ± 20.34	209.00	- 3.52	**0.002**
Weather	Air temperature	Max	22.24 ± 4.05	21.87	- 5.21	**0.000**
		M	14.21 ± 2.78	13.79	- 3.67	**0.000**
		Mdn	14.00 ± 3.46	13.91	- 3.62	**0.000**
		Min	7.26 ± 3.74	6.74	- 2.46	**0.000**
	Atmospheric pressure	IQR	5.69 ± 3.27	5.25	- 0.95	**0.000**
		SD	4.19 ± 1.54	4.18	- 0.50	**0.000**
		M	1014.58 ± 3.07	1014.87	+ 3.01	**0.000**
		Min	1003.67 ± 7.33	1006.00	+ 3.96	**0.000**
	Humidity	Mdn	1015.23 ± 3.61	1016.00	+ 2.91	**0.000**
		Max	1023.41 ± 4.82	1022.00	+ 2.37	**0.000**
		Skew	- 0.36 ± 1.03	- 0.30	+ 0.14	**0.016**
		Min	40.05 ± 9.77	40.00	+ 10.53	**0.000**
		M	65.08 ± 7.53	65.74	+ 5.99	**0.000**
		Mdn	66.15 ± 10.52	66.50	+ 5.97	**0.000**
		SD	14.68 ± 3.68	15.31	- 2.28	**0.000**
		IQR	21.24 ± 9.17	20.00	- 3.98	**0.000**
		Skew	- 0.05 ± 0.60	- 0.10	- 0.09	**0.035**
	Maximum air temperature forecast	Max	23.74 ± 4.14	24.08	- 5.45	**0.000**
		M	15.58 ± 2.80	15.33	- 3.91	**0.000**
		Mdn	15.39 ± 3.52	15.28	- 3.88	**0.000**
		Min	8.69 ± 3.71	8.25	- 2.74	**0.000**
		IQR	5.84 ± 3.20	5.48	- 0.96	**0.000**
		SD	4.28 ± 1.52	4.28	- 0.51	**0.000**
	Minimum air temperature forecast	Max	20.52 ± 4.04	20.69	- 4.94	**0.000**
		M	12.73 ± 2.81	12.35	- 3.43	**0.000**
		Mdn	12.60 ± 3.34	12.62	- 3.38	**0.000**
		Min	5.78 ± 3.95	4.94	- 2.19	**0.000**

		IQR	5.49 ± 3.21	4.77	- 0.94	**0.000**
	Temperature feeling	SD	4.11 ± 1.57	4.13	- 0.51	**0.000**
		Max	21.75 ± 4.18	21.50	- 5.48	**0.000**
		M	13.33 ± 3.03	13.03	- 3.93	**0.000**
		Mdn	13.16 ± 3.79	12.98	- 3.89	**0.000**
		Min	5.61 ± 4.53	5.08	- 2.66	**0.000**
		IQR	5.89 ± 3.64	5.48	- 0.89	**0.000**
		SD	4.45 ± 1.72	4.26	- 0.47	**0.000**
Atmospheric pollutants	AQI	Min	1.07 ± 0.26	1.00	+ 0.13	**0.000**
		SD	0.55 ± 0.16	0.56	- 0.04	**0.011**
		Max	2.80 ± 0.62	3.00	- 0.08	**0.031**
		M	1.87 ± 0.29	1.88	+ 0.05	**0.041**
		IQR	0.57 ± 0.52	0.75	- 0.06	**0.043**
	Ammonia	Skew	1.53 ± 1.06	1.50	- 0.42	**0.000**
		Kurt	2.68 ± 3.89	1.74	- 0.91	**0.000**
		Max	14.18 ± 11.35	11.78	- 1.79	**0.000**
		Min	0.70 ± 0.73	0.56	+ 0.25	**0.001**
		Mdn	2.59 ± 1.94	1.90	+ 0.50	**0.001**
	Carbon monoxide	Min	172.02 ± 21.16	168.56	+ 38.27	**0.000**
		M	232.52 ± 37.62	228.24	+ 54.62	**0.000**
		Mdn	225.09 ± 34.73	220.30	+ 52.57	**0.000**
		IQR	45.16 ± 37.31	35.05	+ 21.67	**0.000**
		Kurt	1.99 ± 4.71	0.49	- 0.67	**0.000**
		Skew	0.98 ± 1.22	0.75	- 0.22	**0.000**
		Max	356.80 ± 130.00	327.11	+ 66.06	**0.000**
		SD	48.09 ± 28.06	42.60	+ 17.66	**0.000**
	Ozone	Max	106.00 ± 18.33	105.86	- 14.08	**0.000**
		M	69.28 ± 13.33	69.87	- 6.95	**0.000**
		Mdn	70.52 ± 16.84	70.10	- 7.19	**0.000**
		SD	22.97 ± 6.60	23.73	- 3.29	**0.000**
		IQR	29.94 ± 14.61	27.54	- 5.18	**0.000**
	Nitric oxide	Kurt	4.87 ± 6.92	2.46	- 1.23	**0.000**
		Skew	1.96 ± 1.37	2.02	- 0.29	**0.000**
		SD	0.87 ± 1.26	0.45	+ 0.71	**0.000**
		M	0.63 ± 1.55	0.30	+ 0.82	**0.000**
		IQR	0.54 ± 0.75	0.26	+ 0.83	**0.000**
		Max	3.46 ± 5.12	1.79	+ 1.47	**0.003**
		Mdn	0.44 ± 2.05	0.11	+ 0.57	**0.017**
		Min	0.03 ± 0.15	0.00	+ 0.10	**0.021**
	Nitrogen dioxide	M	6.04 ± 4.15	4.98	+ 4.76	**0.000**
		Mdn	4.20 ± 3.67	2.74	+ 4.80	**0.000**
		Min	0.86 ± 1.20	0.59	+ 2.46	**0.000**
		Skew	1.38 ± 1.14	1.24	- 0.38	**0.000**
		SD	5.83 ± 4.02	4.89	+ 1.82	**0.000**
		Kurt	2.28 ± 4.54	0.59	- 0.82	**0.000**
		Max	21.46 ± 15.70	18.17	+ 5.34	**0.000**
		IQR	5.48 ± 5.27	4.00	+ 2.32	**0.000**
	PM 2.5	M	5.04 ± 2.72	4.35	+ 3.00	**0.000**
		Mdn	3.73 ± 2.95	2.76	+ 2.93	**0.000**
		Min	0.77 ± 0.91	0.50	+ 1.33	**0.000**
		Skew	1.33 ± 1.09	1.23	- 0.31	**0.000**
		Kurt	2.08 ± 4.30	0.95	- 0.70	**0.000**
		IQR	4.53 ± 3.12	4.05	+ 1.49	**0.000**

		SD	4.84 ± 2.86	4.11	+ 1.35	**0.000**
		Max	18.26 ± 12.60	13.95	+ 3.26	**0.011**
	PM 10	M	6.57 ± 4.04	5.61	+ 3.32	**0.000**
		Mdn	4.84 ± 4.81	3.33	+ 3.38	**0.000**
		Skew	1.35 ± 1.22	1.19	- 0.33	**0.000**
		Min	0.89 ± 1.03	0.58	+ 1.70	**0.000**
		Kurt	2.45 ± 5.54	0.82	- 0.86	**0.000**
		SD	6.50 ± 4.00	5.79	+ 1.11	**0.013**
		IQR	6.48 ± 5.79	5.08	+ 1.08	**0.014**
	Sulfur dioxide	M	1.03 ± 0.72	0.81	+ 1.47	**0.000**
		Mdn	0.71 ± 0.59	0.50	+ 1.32	**0.000**
		Skew	1.50 ± 1.23	1.60	- 0.37	**0.000**
		SD	1.07 ± 0.92	0.78	+ 0.84	**0.000**
		Min	0.16 ± 0.21	0.10	+ 0.48	**0.000**
		Max	4.21 ± 4.08	2.86	+ 2.18	**0.000**
		Kurt	2.95 ± 4.92	1.41	- 0.90	**0.000**
		IQR	0.87 ± 0.80	0.69	+ 1.02	**0.000**
Other	Time zone difference	Kurt	42.03 ± 25.97	43.25	- 13.73	**0.000**
		IQR	0.00 ± 0.00	0.00	+ 0.12	**0.019**
		SD	0.09 ± 0.22	0.00	+ 0.07	**0.028**
	Wear time day percentage	Min	63.46 ± 17.39	57.15	+ 2.62	**0.002**
		Kurt	21.67 ± 16.41	18.25	- 2.50	**0.002**
		Skew	- 4.18 ± 1.97	- 4.14	+ 0.25	**0.011**

Supplementary Material C 3: Temporal Dynamics of the Used Data.

After analysing the trends and values of both the 21 active data outcomes (PROs and PerfROs) and the 40 passive data predictors (TechROs), and before using those in the correlation analysis and modelling phase of this research, one last aspect of that data is important to be addressed. That is the temporal dynamic, which allows us to understand better the seasonality and dynamic evolution of measurements over time. To accomplish such a task, in this subsection, we explore the test-retest reliability and the practice effects for the outcomes of cognition and affective state and explore the autocorrelation of the predictors.

Test-Retest Reliability (Active Data)

The goal of this research is to link the passive data with the active outcomes measured over time, and those PROs and PerfROs being adapted versions of literature-based and already validated scales and performance tests, a first question must be posed. That question is whether the implementation of those scales and tests as a mobile application and its completion in ambulatory settings (as opposite to large-screen lab-based tests) do not affect the consistency of the measurements. For that task, test-retest reliability is a crucial metric in research and assessment that evaluates the consistency of a test's results over time. It indicates to what extent a measurement tool produces the same output when executed under the same internal and external conditions.

Thus, to assess reliability of the used ambulatory versions of the measurement tools: (1) PROs designed to evaluate cognition or affective state components that require extended periods to change significantly (e.g., cognitive decline) are expected to indicate a high-reliability score, (2) PROs reflecting elements with higher volatility in shorter periods (e.g., stress) are expected to indicate a lower reliability score, and (3) PerfROs assessing cognitive performance are expected to show the lowest? reliability scores in the group of all active data outcomes, as fluctuations in everyday life are frequent—confirming the need for frequent cognitive performance assessment in healthy individuals.

The results of this test are to be read according to the *Intraclass Correlation Coefficient* (ICC). We interpret them below, following the guidelines by Koo and Li [12]: poor if below 0.50, moderate if between 0.50 and 0.75, good if between 0.75 and 0.90, and excellent reliability if above 0.90. Table S16 contains the results obtained across all four waves of active data outcomes, ordered from the highest to the lowest ICC. From its contents, we can extract at least two broad insights: (1) only PerfROs show poor test-retest reliability, (2) the only PerfROs not in the poor results group are tapping and typing speed, measuring motor speed and digital typing skills, respectively, and (3) all PROs recorded a moderate to good test-retest. In conclusion, the PROs we used to cognition and assess affective states with mobile and ambulatory tools such as smartphones showed an overall good reliability of their outputs. More specifically, a trend is apparent when crossing the ICC of each PRO and the recall period of each of them, revealing that the reliability achieved so far with four waves seems to be linked to the defined recall for a given outcome, with bigger periods linking to a higher ICC value. As for the PerfROs, by looking at the majority of those, we can see that memory is the most stable measurement in that group, followed by reasoning, verbal fluency, and attention. That may also be due to the time for memory and other measured mechanisms of cognition to change over time, some being more susceptible to the short-term influence of external factors than others (e.g., weather, sleep).

Table S17 - Results of the test-retest reliability analysis of each outcome.

**Active data outcome**	**ICC (↓)**	**Type**	**Recall period**
Memory complaints	0.82 (**Good**)	PRO	Not specified
Cognitive decline	0.79 (**Good**)	PRO	10 years
Hostility	0.75 (**Good**)	PRO	1 week
Tapping speed	0.75 (**Good**)	PerfRO	Not applicable
Stress	0.67 (**Moderate**)	PRO	1 month
Typing speed	0.66 (**Moderate**)	PerfRO	Not applicable
Anxiety	0.65 (**Moderate**)	PRO	1 week

Positive affect	0.63 (**Moderate**)	PRO	1 week
Depression	0.53 (**Moderate**)	PRO	1 week
Negative affect	0.51 (**Moderate**)	PRO	1 week
Working memory	0.47 (**Poor**)	PerfRO	Not applicable
Prospective memory	0.41 (**Poor**)	PerfRO	Not applicable
Short-term memory	0.40 (**Poor**)	PerfRO	Not applicable
Long-term memory	0.38 (**Poor**)	PerfRO	Not applicable
Inductive reasoning	0.36 (**Poor**)	PerfRO	Not applicable
Verbal fluency 1	0.27 (**Poor**)	PerfRO	Not applicable
Attention	0.23 (**Poor**)	PerfRO	Not applicable
Verbal fluency 2	0.22 (**Poor**)	PerfRO	Not applicable
Cognitive flexibility	0.21 (**Poor**)	PerfRO	Not applicable
Processing speed	0.12 (**Poor**)	PerfRO	Not applicable
Inhibitory control	- 0.06 (**Poor**)	PerfRO	Not applicable

Practice Effects (Active Data)

Practice effects may pose a threat to research like ours, in which participants are tested multiple times for the same outcome to understand its evolution over time. Therefore, we ran a practice effects analysis on all the PROs and PerfROs used as outcomes of cognition and affective state (21). Practice effects might justify, for example, improved speed on the completion of tasks and enhanced performance by skill retention and remembering the previous answers. Given its different nature and way of evaluating the participants, we expect PerfROs to display the highest practice effects over waves, while a small effect is also to be expected to be observed on the PROs as individuals can still remember what they answered to each question after each wave is finished (around three months).

We employed the T-test and the Wilcoxon signed-rank test to compare the values of each outcome between two consecutive waves. The first test is appropriate for normally distributed continuous data, while the second is its non-parametric alternative. We assume a practice effect whenever the p-value of any of those tests is significant. Furthermore, when that is the case, we interpret the difference between the mean values of the two waves across all participants. Table S17 includes the results of this analysis for all the PROs and PerfROs. The p-values of both tests are displayed in columns “T p” and “W p,” which stand for T-test p-value and Wilcoxon signed-rank p-value, respectively. The difference between the wave’s mean values is provided in column “ΔM”, in which a positive value means an increase from the first wave to the second (from Wave 1 to Wave 2 in the “Wave 1 to Wave 2” results).

Table S18 - Practice effects for each outcome used in this research, ordered according to the results of the test-retest analysis. "T p," "W p," and " ΔM" mean the p-values for the T-test and Wilcoxon signed rank and the difference between the mean of waves, respectively.

**Active data outcome**	**Type**	**Wave 1 to Wave 2**			**Wave 2 to Wave 3**			**Wave 3 to Wave 4**		
		**T p**	**W p**	**ΔM**	**T p**	**W p**	**ΔM**	**T p**	**W p**	**ΔM**
Memory cogn.	PRO	0.59	0.58	-0.32	0.08	0.10	1.23	0.06	0.07	-1.34
Cognitive dec.	PRO	0.44	0.36	0.02	0.90	0.92	0.00	0.45	0.46	-0.02
Hostility	PRO	0.34	0.34	0.06	0.40	0.40	-0.06	0.84	0.83	0.01
Tapping speed	PerfRO	0.05	0.02	-15.82	0.89	0.94	1.03	0.05	0.04	-14.1
Stress	PRO	0.01	0.04	0.13	0.16	0.19	-0.07	0.43	0.63	0.04
Typing speed	PerfRO	0.42	0.45	0.03	0.26	0.21	0.06	0.81	0.69	-0.01
Anxiety	PRO	0.14	0.13	-0.38	0.13	0.16	-0.35	0.07	0.06	0.44
Positive affect	PRO	0.62	0.71	-0.35	0.06	0.02	1.50	0.18	0.13	-0.91
Depression	PRO	0.48	0.50	0.18	0.05	0.08	-0.62	0.13	0.29	0.44
Negative affe.	PRO	0.23	0.51	0.84	0.08	0.18	-1.14	0.43	0.39	0.49
Working mem.	PerfRO	0.03	0.02	0.73	0.00	0.00	-1.05	0.01	0.00	1.07
Prospecti. m.	PerfRO	0.04	0.04	0.12	0.00	0.00	0.19	0.09	0.09	-0.09
Short-term m.	PerfRO	0.00	0.00	1.07	0.21	0.16	-0.32	0.00	0.00	1.37

Long-term m.	PerfRO	0.01	0.01	0.64	0.63	0.48	-0.12	0.00	0.00	1.40
Inductive reas.	PerfRO	0.77	0.78	0.07	0.44	0.29	0.19	0.48	0.56	0.16
Verbal fluen. 1	PerfRO	0.10	0.08	1.11	0.00	0.00	2.51	0.00	0.00	-1.44
Attention	PerfRO	0.70	0.99	-56.27	0.49	0.42	-120.5	0.29	0.42	143.7
Verbal fluen. 2	PerfRO	0.57	0.95	0.37	0.13	0.11	-0.72	0.02	0.04	1.13
Cognitive flex.	PerfRO	0.85	0.91	0.21	0.10	0.09	-1.75	0.40	0.81	0.61
Processing sp.	PerfRO	0.82	0.87	0.12	0.45	0.05	-0.43	0.75	0.37	0.22
Inhibitory con.	PerfRO	0.07	0.34	-90.68	0.05	0.06	101.10	0.70	0.04	57.38

Overall, we can conclude that:

1. Three out of eight PROs displayed practice effects at least for one pair of waves, but no PRO showed them across all waves. The reported stress levels revealed a practice effect between the two first waves, which can be explained by the fact that not all participants had around three months between assessments. That is, as the enrolment was made across Wave 1, some individuals had only one month between the two stress assessments. For that reason, the recall period of one month overlapped between assessments, leading to similar results for some participants, which here is interpreted as a learning effect for those measurements.
2. Out of the 13 PerfROs, only one of them showed practice effects over all waves (working memory), although the signal of mean differences inverted over time (increased scoring between waves 1 and 2, decreased between waves 2 and 3, and increased again between waves 3 and 4). Seven others showed practice effects for at least one pair of consecutive waves. However, only three displayed them from wave 2 to wave 4, for which all except one had inversed mean differences signals over time. That may indicate that, despite obtaining a discernible value for at least one of the tests, the practice effects are inconsistent for those measures, leading the participants to increase performance and decrease.

Autocorrelation Analysis (Passive Data)

Since passive data is recorded continuously over successive waves, data points collected close in time are not independent of each other. Ignoring such temporal dependencies could lead to a bias and misinterpretation of the modelling results presented in this manuscript. Therefore, autocorrelation was examined to understand better the temporal structure of the wearable data and ensure the validity of the subsequential analyses.

Autocorrelation can range between -1 and +1, the former meaning perfect negative correlation and the latter a perfect positive one. A perfect positive case means high-value consistency from one day to the next (e.g., a very stable variation of HR levels or total steps). Contrarily, a perfect negative correlation indicates an alternation of values over time (e.g., high HR on a day, low HR on the next day, and high HR again the day after). Finally, when obtaining an autocorrelation next to 0 for a specific type of data, we can infer that there is no correlation between the values over the consecutive days—values from one day do not predict the following ones (e.g., HR variable due to external factors such as stress, environmental changes).

As shown in Table S18, after ordering the results from the highest to the lowest autocorrelation across all waves, there are seven out of 25 wearable-derived outcomes above around 0.40 of autocorrelation. The highest autocorrelation is present in the wear time across days, showing that participants' wearing habits are somehow constant over time. Following that, the second highest value is for the mean HR of 24h, meaning that HR variability across waves is not frequently caused by external factors but rather predictable for the average participant's data. All the remaining five top autocorrelations are between 0.39 and 0.43 and link to physical activity levels (steps, distance, calories, exercise rates). Overall, these results across waves show higher stability in the physical activity levels compared to HR and sleep measures. Furthermore, when analysing the trend of those seven top autocorrelated

outcomes in each of the four waves, all of them decrease their autocorrelation drastically from wave 1 to 2, then slightly to wave 3.

Table S19 - Autocorrelation per wearable-derived TechRO across waves and in each wave separately. Ordered according to the "all waves" score decreasing. Units are specified in Table S9.

**Wearable outcome**	**All waves (↓)**	**Wave 1**	**Wave 2**	**Wave 3**	**Wave 4 (incomplete)**
Wear time dur. %	**0.82 ± 0.22**	0.70 ± 0.38	0.29 ± 0.33	0.25 ± 0.27	0.33 ± 0.38
24h HR mean	**0.73 ± 0.29**	0.70 ± 0.32	0.35 ± 0.31	0.31 ± 0.29	0.32 ± 0.34
Steps	**0.43 ± 0.19**	0.49 ± 0.26	0.25 ± 0.22	0.18 ± 0.23	0.22 ± 0.27
Distance	**0.42 ± 0.19**	0.48 ± 0.26	0.25 ± 0.22	0.18 ± 0.23	0.21 ± 0.28
Active calories	**0.40 ± 0.19**	0.46 ± 0.27	0.24 ± 0.23	0.17 ± 0.23	0.20 ± 0.28
Step frequency	**0.40 ± 0.18**	0.47 ± 0.26	0.23 ± 0.21	0.18 ± 0.22	0.20 ± 0.25
Distance rate	**0.39 ± 0.18**	0.46 ± 0.27	0.22 ± 0.21	0.18 ± 0.22	0.19 ± 0.25
Sleep. HR M	0.34 ± 0.25	0.22 ± 0.27	0.24 ± 0.25	0.29 ± 0.28	0.22 ± 0.24
Sleep. HR Min	0.28 ± 0.24	0.19 ± 0.25	0.19 ± 0.24	0.19 ± 0.28	0.13 ± 0.25
24h HR Mdn	0.23 ± 0.22	0.18 ± 0.24	0.24 ± 0.22	0.16 ± 0.20	0.18 ± 0.21
Sleep. HR Max	0.15 ± 0.25	0.08 ± 0.24	0.08 ± 0.22	0.13 ± 0.27	0.05 ± 0.22
Light sleep durat.	0.12 ± 0.14	0.09 ± 0.15	0.08 ± 0.16	0.07 ± 0.16	0.06 ± 0.24
Time zone differ.	0.11 ± 0.55	- 0.14 ± 0.29	- 0.16 ± 0.30	0.56 ± 0.47	0.25 ± 0.64
24h HR Min	0.11 ± 0.13	0.05 ± 0.17	0.08 ± 0.14	0.05 ± 0.13	0.08 ± 0.16
Wake-up count	0.07 ± 0.12	0.02 ± 0.16	0.08 ± 0.14	0.03 ± 0.16	0.02 ± 0.21
Sleep efficiency	0.06 ± 0.11	0.00 ± 0.16	0.06 ± 0.14	0.01 ± 0.19	0.00 ± 0.23
WASO	0.05 ± 0.11	0.01 ± 0.14	0.07 ± 0.16	0.03 ± 0.16	0.01 ± 0.21
24h HR SD	0.05 ± 0.12	0.01 ± 0.14	0.02 ± 0.19	0.01 ± 0.13	0.04 ± 0.20
Time in bed	0.04 ± 0.14	0.02 ± 0.16	0.01 ± 0.16	0.00 ± 0.17	0.03 ± 0.25
Wake-up latency	0.04 ± 0.10	0.02 ± 0.15	0.04 ± 0.14	0.01 ± 0.11	- 0.03 ± 0.20
Total sleep time	0.03 ± 0.13	0.01 ± 0.16	0.00 ± 0.17	- 0.01 ± 0.16	0.03 ± 0.25
24h HR Max	0.03 ± 0.09	0.02 ± 0.13	0.00 ± 0.16	0.01 ± 0.12	- 0.01 ± 0.19
Sleep score	0.03 ± 0.13	0.00 ± 0.15	0.00 ± 0.18	- 0.02 ± 0.14	0.01 ± 0.24
Deep sleep durat.	0.03 ± 0.14	- 0.01 ± 0.17	0.01 ± 0.17	- 0.05 ± 0.14	- 0.02 ± 0.23
Sleep latency	0.01 ± 0.09	- 0.01 ± 0.10	- 0.01 ± 0.09	0.01 ± 0.14	- 0.04 ± 0.14

The highest autocorrelations (in decreasing order) were found for the wear time across days, followed by the mean HR of 24h, which is followed by autocorrelation between 0.39 and 0.43 for measurements linking to physical activity levels (steps, distance, calories, exercise rates). Overall, these results across waves show higher stability in the physical activity levels compared to HR and sleep measures. Furthermore, when analysing the trend of those seven top autocorrelated outcomes in each of the four waves, all of them decrease their autocorrelation drastically from wave 1 to 2, then slightly to wave 3. That means that, although overall these seven outcomes look somewhat stable, only in wave 1 we should expect such stability.

Correlation between Active and Passive Data

As detailed earlier in this manuscript, eight features were extracted to summarize the information of each TechRO over each wave for each data representation. We then used three correlation coefficient analyses to select the most correlated metric to use when modelling each of the PROs and PerfROs. This subsection summarizes the features selection across models for each coefficient used. An exhaustive listing and discussion of the specific correlations between each pair of predictor's passive metrics and the active outcome to be predicted will be the focus of a future publication and are, therefore, out of the scope of this article.

Standardization of Data (Active and Passive)

The correlation analysis was run in two distinct ways: (1) with the original scale of both active and passive data, and (2) with the data z-score normalization (also referred to as standardization). We did so for comparison of results and chose the standardized version of data for the subsequent steps of analysis, opting for the most robust and comparable interpretation across variables with different scaling. Table S19 shows the mean absolute correlation coefficients obtained.

Table S20 - Mean absolute correlation coefficients with and without data standardization for each representation type and coefficient.

Repres. type	Standardization	M Abs. P. corr.	M Abs. S. corr.	M Abs. K. corr.
Levels	No	0.070 (± 0.065)	0.076 (± 0.075)	0.056 (± 0.056)
	**Yes**	**0.078 (± 0.075)**	**0.078 (± 0.076)**	**0.057 (± 0.056)**
ΔABS	No	**0.068 (± 0.057)**	0.065 (± 0.054)	0.044 (± 0.038)
	**Yes**	0.062 (± 0.048)	**0.067 (± 0.056)**	**0.046 (± 0.039)**
Δ%	No	0.065 (± 0.053)	0.064 (± 0.052)	0.043 (± 0.037)
	**Yes**	**0.066 (± 0.049)**	**0.066 (± 0.055)**	**0.045 (± 0.039)**

References

1. Zigmond, A. S. & Snaith, R. P. The Hospital Anxiety and Depression Scale. *Acta Psychiatr Scand* **67**, 361–370 (1983).

2. Jorm, A. F. & Jacomb, P. A. The Informant Questionnaire on Cognitive Decline in the Elderly (IQCODE): Socio-demographic correlates, reliability, validity and some norms. *Psychol Med* **19**, 1015–1022 (1989).

3. Buss, A. H. & Durkee, A. An inventory for assessing different kinds of hostility. *J Consult Psychol* **21**, 343–349 (1957).

4. Smith, G., Della Sala, S., Logie, R. H. & Maylor, E. A. Prospective and retrospective memory in normal ageing and dementia: A questionnaire study. *Memory* **8**, 311–321 (2000).

5. Watson, D., Clark, L. A. & Tellegen, A. Development and Validation of Brief Measures of Positive and Negative Affect: The PANAS Scales. *J Pers Soc Psychol* **54**, 1063–1070 (1988).

6. Cohen, S., Kamarck, T. & Mermelstein, R. A global measure of perceived stress. *J Health Soc Behav* **24**, 385–396 (1983).

7. Deary, I. J., Liewald, D. & Nissan, J. A free, easy-to-use, computer-based simple and four-choice reaction time programme: The Deary-Liewald reaction time task. *Behav Res Methods* **43**, 258–268 (2011).

8. Tombaugh, T. N. Trail Making Test A and B: Normative data stratified by age and education. *Archives of Clinical Neuropsychology* **19**, 203–214 (2004).

9. Haas, M., Scheibe, S. & Khawli, E. El. Online Assessment of Cognitive Functioning Across the Adult Lifespan Using the eCOGTEL: A Reliable Alternative to Laboratory Testing. https://www.researchgate.net/publication/356407525.

10. Eriksen, B. A. & Eriksen, C. W. Effects of noise letters upon the identification of a target letter in a nonsearch task. *Percept Psychophys* **16**, 143–149 (1974).

11. Ward, T. *et al.* Finger-Tapping Test. in *Encyclopedia of Autism Spectrum Disorders* 1296–1296 (Springer, New York, NY, 2013). doi:10.1007/978-1-4419-1698-3_343.

12. Koo, T. K. & Li, M. Y. A Guideline of Selecting and Reporting Intraclass Correlation Coefficients for Reliability Research. *J Chiropr Med* **15**, 155 (2016).
